# KLF5 inhibition overcomes oxaliplatin resistance in patient-derived colorectal cancer organoids by restoring apoptotic response

**DOI:** 10.1038/s41419-022-04773-1

**Published:** 2022-04-05

**Authors:** Xiaohui Shen, Yuchen Zhang, Zhuoqing Xu, Han Gao, Wenqing Feng, Wenchang Li, Yiming Miao, Zifeng Xu, Yaping Zong, Jingkun Zhao, Aiguo Lu

**Affiliations:** 1 Department of General Surgery, Ruijin Hospital, Shanghai Jiaotong University School of Medicine, Shanghai, P. R. China; 2 Shanghai Institute of Digestive Surgery, Shanghai, P. R. China; 3 Shanghai Minimally Invasive Surgery Center, Ruijin hospital, Shanghai Jiaotong University School of Medicine, Shanghai, P. R. China

**Keywords:** Cancer therapeutic resistance, Colorectal cancer, Cancer models

## Abstract

Oxaliplatin resistance is a major challenge in the treatment of colorectal cancer (CRC). Many molecular targeted drugs for refractory CRC have been developed to solve CRC drug resistance, but their effectiveness and roles in the progression of CRC and oxaliplatin resistance remain unclear. Here, we successfully constructed CRC PDOs and selected the Kruppel-like factor 5 (KLF5) inhibitor ML264 as the research object based on the results of the in vitro drug screening assay. ML264 significantly restored oxaliplatin sensitivity in CRC PDOs by restoring the apoptotic response, and this effect was achieved by inhibiting the KLF5/Bcl-2/caspase3 signaling pathway. Chromatin immunoprecipitation (ChIP) and luciferase reporter assays verified that KLF5 promoted the transcription of Bcl-2 in CRC cells. KLF5 inhibition also overcame oxaliplatin resistance in xenograft tumors. Taken together, our study demonstrated that ML264 can restore oxaliplatin sensitivity in CRC PDOs by restoring the apoptotic response. KLF5 may be a potential therapeutic target for oxaliplatin-resistant CRC. PDOs have a strong potential for evaluating inhibitors and drug combination therapy in a preclinical environment.

## Introduction

CRC is one of the most common types of malignant tumors of the digestive system [1]. The standard treatments for CRC include surgical resection, chemotherapy, and radiation. The platinum anti-tumor agent oxaliplatin is widely used as a first-line chemotherapy drug, typically combined with 5-fluorouracil (5-FU) and folinic acid for the treatment of CRC. Although the clinical application of oxaliplatin improved the prognosis of CRC patients to a certain extent, drug resistance often occurs after short-term use of oxaliplatin, which leads to therapeutic failure and poor prognosis [2]. Modern molecular targeted therapy for metastatic CRC is considered to be one of the effective ways to solve CRC drug resistance, which is also the focus of current oncology research [3, 4]. Organoids are micro-organs with three-dimensional structures formed by stem cells under special culture conditions in vitro [5]. Currently, Patient-derived organoids (PDOs) have been constructed from various carcinomas, such as gastric [6], colorectal [7], pancreatic [8], breast [9], and ovarian cancers [10]. PDOs retain the characteristics of the primary tumor, so they are particularly suitable for personalized drug testing and high-throughput drug screening in vitro [11].

KLF5, a stem-related transcription factor, plays an important role in tumor cell proliferation, survival, apoptosis, and drug sensitivity [12]. KLF5 is generally considered protumorigenic, and it has been reported to be highly expressed in CRC and promotes the proliferation and progression of CRC cells [13, 14]. The drug resistance of tumor cells or tumor stem cells may be related to their ability to adaptively reprogram in response to drug treatment. Previous studies have suggested that KLF5 may be involved in this process [15, 16].

Inouea et al. reported a new method to generate PDOs from primary CRC tissues, in which the partially dissociated cell clusters quickly formed spheroids, named cancer tissue-originated spheroids (CTOSs) [17]. CTOSs are composed of highly purified cancer cells that retain the characteristics of parental tumors and grow stably in vitro. In this study, we employed the CTOS method to generate PDOs to further explore the potential molecular mechanisms of oxaliplatin resistance in CRC, including conducting oxaliplatin sensitivity assays and drug screening assays in vitro. We first successfully constructed PDOs derived from tumor tissues of CRC patients in vitro and conducted a drug screening assay to find that ML264, a KLF5 inhibitor [14], could restore oxaliplatin sensitivity in CRC PDOs. In addition, we found that the sensitivity of CRC organoids to oxaliplatin is negatively correlated with the expression of KLF5, and KLF5 can inhibit the apoptosis of CRC cells induced by oxaliplatin. Finally, we confirmed that the KLF5 inhibitor, ML264, can enhance the efficiency of oxaliplatin therapy in CRC in vivo and in vitro. These findings indicate that KLF5 participates in the resistance mechanism of CRC to oxaliplatin by inhibiting cell apoptosis. In addition, our study shows the strong potential of PDOs in evaluating inhibitor and drug combination therapy in a preclinical environment.

## Methods

### Patients and specimens

The specimens of 14 patients were collected after obtaining the informed consent from the Biomedical Ethics Committee of Ruijin Hospital (Table 1, S1). All these patients were diagnosed as CRC pathologically and accepted laparoscopic surgery in Minimally Invasive Surgery Centre, Ruijin Hospital, Shanghai Jiaotong University. All the patients who had received preoperative treatment such as radiation or chemotherapy were excluded. Pathological staging of CRC tumor was performed in accordance to the TNM classification.

**Table 1:** Supplementary clinical information of PDOs donor.

Sample ID	Tumor stage	Type of treatments	Clinical outcome	Mutation		
				KRas	Nras	Braf
P1	T3N0M0	Surgery	Survival	Wild	Wild	Wild
P2	T3N2M0	Postoperative chemotherapy (XELOX)	Survival	Wild	Wild	Wild
P3	T3N0M0	Surgery	Survival	Not detected	Not detected	Not detected
P4	T3N1M1	mFOLFOX6 + cetuxima, liver metastases resection, postoperative chemotherapy (XELOX)	Recurrence	Wild	Wild	Wild
P5	T3N1M1	FOLFOX, liver metastases resection, Postoperative chemotherapy (XELOX)	Survival	exon 2 mutation	Wild	Wild
P6	T4N1M1	mFOLFOX6, liver metastases resection, Postoperative chemotherapy (XELOX)	Recurrence	exon 2 mutation	Wild	Wild
P7	T3N1M0	Postoperative chemotherapy (XELOX)	Survival	Wild	Wild	Wild
P8	T3N1M0	Postoperative chemotherapy (XELOX)	Survival	Not detected	Not detected	Not detected
P9	T3N0M0	Surgery	Survival	Wild	Wild	Wild
P10	T3N0M0	Surgery	Survival	Not detected	Not detected	Not detected
P11	T3N1M0	Postoperative chemotherapy (XELOX)	Survival	Wild	Wild	Wild
P12	T3N1M0	Postoperative chemotherapy (XELOX)	Survival	exon 2 mutation	Wild	Wild
P13	T3N1M0	Postoperative chemotherapy (XELOX)	Survival	Not detected	Not detected	Not detected
P14	T3N1M0	Postoperative chemotherapy (XELOX)	Survival	Not detected	Not detected	Not detected

### Specimen processing and tumor cell preparation

Specimen processing and tumor cell preparation were performed as previously described [17]. Surgical specimens were washed with PBS containing penicillin-streptomycin solution and minced into meat emulsion using tweezers and disposable surgical blades. Tissue fragments were transferred to a 50 ml centrifuge tube and mixed with 0.5 mg/ml type IV collagenase (Sigma, USA) in DMEM at 37 °C for 1 h until fully digested. The digested tissue suspension was filtered through 500 μm and 100 μm cell strainers (BD Falcon, USA) in sequence to remove residual tissue. The filtrate was then re-filtered through a 40 μm cell strainer, and the cell clusters retained in the strainer were collected and washed twice with PBS. Cell clusters were transferred to the stem cell culture medium (DMEM/F12 medium with glutamine, non-essential amino acids, 8 ng/mL bFGF, 2-mercaptoethanol, serum substitute, and penicillin-streptomycin solution) (Cyagen, China) and cultured at 37 °C, 5% CO2, and 20% O2. After 24 h of cultivation, CTOSs with obvious spherical structures and smooth surfaces were formed.

### The cultivation and expansion of PDOs

Once PDOs were formed, the growth factors required for CRC organoid culture were added to the stem cell medium, including 50 ng/mL EGF (PeproTech, USA), 500 nM A83-01 (AbMole, USA), 50 ng/mL Noggin (PeproTech, USA), 3 μM SB202190 (AbMole, USA), 10 nM prostaglandin E2 (AbMole, USA), and 1X B27 (Gibco, USA). A 23-gauge needle was used to tear the PDOs for expansion. For 3D culture, the PDOs were embedded in Matrigel (BD, USA) and overlaid with organoid culture medium. TrypLE™ Express Enzyme (Thermo Scientific, USA) was used to digest the Matrigel to release PDOs. More information about the Reagents was listed in Table S2.

### Flow cytometry analysis

PDOs were collected and washed with PBS, and digested into single cell suspension using 0.25% trypsin/EDTA. The cells were washed with PBS, and then incubated with fluorescent-conjugated primary antibodies in PBS containing 0.5% BSA at 4 °C in the dark for 30 min. Antibodies used including EpCAM, CEA CAM1, CD31, CD45, CD133, CD166. More information about the antibody was listed in Table S3. Samples were detected using flow cytometry (BD Biosciences, USA) and analyzed by FlowJo software (Tree Star).

### Histological staining

Tissues and PDOs were fixed in 4% neutral buffered formalin and embedded in paraffin. H&E staining and immunohistochemical staining were performed on the slices. Antibodies of IHC analysis included Ki-67, EpCAM, MUC2, α-SMA, CD68. More information about the antibody was listed in Table S4. TUNEL apoptosis detection kit was used according to the manufacturer’s instructions. Positive cells were scored irrespective of the intensity of staining. The percentage of positive cells was scored semi-quantitatively by two independent individuals.

### Immunofluorescence assays and confocal microscopy

Immunofluorescence assays were performed as previously described [18]. PDOs were collected and fixed with 4% paraformaldehyde for 15 min. After washing with PBS, PDOs were permeabilized with 0.1% Triton X-100 at room temperature for 5 min. Then PDOs were washed with PBS and blocked with 5% BSA for 1 h at room temperature. PDOs were incubated with primary antibodies overnight at 4 °C, including E-Cadherin, β-catenin, Ki-67 and EpCAM. Details of the antibody were listed in Table S5. After that, PDOs were washed and incubated with Alexa Fluor 488 or Alexa Fluor 555 Secondary Antibody (Abcam, UK) at 37 °C for 2 h. After washing with PBS, diamidino phenylindole (DAPI, Santa Cruz, USA) was used to counterstain the nucleus. The results were visualized using a laser scanning confocal microscope (Zeiss, LSM510).

### Apoptosis detection

Flow cytometric assays of apoptosis were performed as previously described [19]. Annexin V-FITC Apoptosis Detection Kit (BD Pharmingen, USA) was used to detect cell surface Annexin V expression and propidium iodide (PI) uptake by flow cytometry. Cells were collected, washed twice with ice-cold PBS, and suspended in 100 μl binding buffer. Using 3 µl Annexin V-FITC and 5 µl PI to stain cells in the dark at room temperature for 15 min. Apoptosis was analyzed by flow cytometry using the FACS Calibur system (BD Biosciences, USA). According to the manufacturer’s instructions, using TUNEL Apoptosis Detection Kit (Beyotime, China) to stain PDOs, and detecting apoptosis by fluorescence microscope. GreenNuc™ Caspase-3 Assay Kit (Beyotime, China) was used to detect Caspase-3/7 activity in PDOs according to the manufacturer’s instructions. Using Ac-DEVD-CHO, a reversible inhibitor of Caspase-3/7, as a negative control. The results were visualized by fluorescence microscope.

### Animal research

Four-week-old male BALB/c nude mice were purchased from the Shanghai Institute of Zoology, Chinese Academy of Sciences. All experiments were performed in accordance with the official recommendations of the Chinese Zoological Society, and animals were cared for humanely in accordance with the standards outlined in the “Guidelines for the Care and Use of Laboratory Animals.” CRC cells were injected subcutaneously into the flanks of the nude mice. The drug dose for the mice experiments was set up according to the manufacturer’s protocol, including ML264 25 mg/kg (TargetMol, China) and oxaliplatin 5 mg/kg (TargetMol, China). When the tumors reached approximately 5 mm in diameter, all mice were randomly divided into four groups (3 in each group: Group 1, vehicle-only solution; Group 2, ML264 25 mg/kg; Group 3, oxaliplatin 5 mg/kg; Group 4, oxaliplatin 5 mg/kg + ML264 25 mg/kg. These compounds were dissolved in NS (physiological saline solution) and administered intraperitoneally. The dosing regimen was oxaliplatin once a week, ML264 twice a week, and each treatment regimen lasted for a duration of 14 days. A Vernier caliper was used to measure the tumor size and the data was recorded twice a week. Three days after the last injection, the animals were sacrificed by cervical decapitation, and tumors were excised and retained for further analyses. Samples were prepared for western blot and histological staining.

### Evaluation of PDO growth

PDO growth was evaluated as previously described [17]. PDOs were evaluated after culturing in Matrigel for 1 week. The PDO growth ratio was calculated as follows: (major axis length) × (minor axis length) after cultivation / (major axis length) × (minor axis length) before cultivation. CellTiter-Glo® luminescent cell viability assay (Promega, USA) was also used to measure the viability of the organoids according to the manufacturer’s instructions (Fig. S2A, B).

### Drug sensitivity analysis

Stable-growing CRC PDOs were used for drug sensitivity testing. Approximately 50 PDOs were embedded in Matrigel, plated in 96-well plates, and overlaid with organoid culture medium. The oxaliplatin concentrations in the drug sensitivity analysis were chosen based on the clinically relevant concentrations of oxaliplatin. Thus, PDOs were treated with oxaliplatin from 0 to 100 μM, and DMSO was added to the culture as a solvent control. The dose of ML264 for cell experiments was set at 10 μM, according to the manufacturer’s protocol. After seeding the tumor spheroids, the initial image and data were recorded, and the indicated drugs were added. The plate was incubated at 37 °C and 5% CO2 for seven days, and images were captured on days 1, 3, 5, and 7 to evaluate PDO growth and CellTiter-Glo® luminescent cell viability assay was used to calculate the growth ratio (Fig. S2C, D, E). Curve fitting of drug sensitivity data was conducted as previously described [20], and the data were expressed as a percentage of growth starting from 0 μM. The area under the curve (AUC) was calculated and normalized as previously described [21]. An half maximal inhibitory concentration (IC50) value of 4 μM was selected as the cut-off value between the oxaliplatin-sensitive vs. resistant model based on the dose-response curves and AUC values of different PDOs (Fig. S1E, F, G).

### Drug screening assay

We selected 60 FDA-approved anticancer drugs and small molecule compounds (Supplementary Data 2 and 3) from the Anti-cancer Compound Library (Supplementary Data 1) of Target Mol, which have been reported to be used for drug screening assays for digestive system tumors [4, 7]. The oxaliplatin-resistant PDOs were used for the drug screening assay and were divided into two groups (Group 1: the selected drug was used as a monotherapy; Group 2: the selected drug was combined with oxaliplatin as multi-therapy). The concentration of the compounds was 3 μM. The plates were incubated at 37 °C and 5% CO2 for seven days. The PDO growth was observed and recorded every day, and the growth rate was calculated.

### Cell lines

The human CRC cell lines used in our study were purchased from the American Type Culture Collection (ATCC, USA). All these cells were cultured in RPMI-1640 medium with 10% fetal bovine serum (FBS), penicillin (107 U/l), and streptomycin (10 mg/l) and incubated at 37 °C and 5% CO2. Cells used in this study were authenticated by STR profiling according to the cell bank.

### RNA extraction and quantitative real-time PCR (qRT-PCR)

RNA extraction and qRT-PCR were performed as previously described [22]. Total RNA of PDOs was extracted using RNAprep pure Micro Kit (Tiangen, China) according to the manufacturer’s instructions. Total RNA was reversed to cDNA using HiScript III RT SuperMix (Vazyme, China) and qPCR was performed using SYBR Green (Vazyme, China) according to the manufacturer’s instructions. The primers of qPCR were purchased from Genewiz, China and the sequences were listed in Table S6. GAPDH was used as control. The relative expression ratio of mRNAs in each group was calculated by the 2^−ΔΔCT^ method.

### Protein extraction and Western blotting

Protein extraction and western blot analysis was performed as previously described [18]. For CRC organoids, about 1000 PDOs were collected for protein extraction. For adherent CRC cells, cells were collected at a 70–80% confluence, and total protein was extracted by RIPA (Solarbio, China) in the presence of Protease Inhibitor and Protein Phosphatase Inhibitor Cocktail (APExBIO, USA). The concentration of total protein lysate was quantified using BCA Protein Assay Kit (ThermoFisher, USA). Proteins were separated by SDS-PAGE and transferred to a PVDF membrane (Tanon, China). Antibodies of western blotting included KLF5, Cleaved Caspase-3, Caspase-3, Bcl-2, Bax and GAPDH. Details of the antibody were listed in Table S7. Goat anti-rabbit or goat anti-mouse HRP-conjugated IgG was used as the secondary antibody (proteintech, USA), and samples were incubated at room temperature for 2 h. The ECL chemiluminescence agent (Millipore, USA) was used to visualize the membrane. The image was captured by Tanon Chemiluminescence Imaging System (Tanon, China). GAPDH was used as the internal control.

### siRNA knockdown

CRC cells were transiently transfected with BCL2 or Bax siRNA (TSINGKE, China) using Lipofectamine™ 3000 Transfection Reagent (Invitrogen, USA) according to the manufacturer’s instructions. Western blot was used to test the downregulation efficiency of siRNA. The targeting site sequences were listed in Table S8.

### Lentivirus vectors and shRNA construction

The lentiviral vectors LV5-EF1a-GFP/Puro-KLF5, LV3-pGLV-h1-GFP/puro-sh-KLF5, LV5-EF1a-GFP/Puro-Vector and LV3-pGLV-h1-GFP/puro-sh-NC were constructed by the Shanghai GenePharma Corporation (China). The sequences of sh-KLF5 are shown in Table S9. The overexpression and interfering effects of these vector/shRNA were evaluated by westernblot.

### Chromatin immunoprecipitation (CHIP)

ChIP assays were performed as previously described [23]. SimpleChIP® Plus Enzymatic Chromatin IP Kit (CST, USA) was used according to the manufacturer’s instructions. The presence of predicted transcription factor binding regions pulled by antibodies was examined by qPCR. The primers of qPCR were purchased from Genewiz, China and the sequences were listed in Table S6.

### Luciferase reporter assay

The luciferase reporter assay was performed as described previously [24]. The BCL2 promoter was cloned into the pGL3-Basic luciferase plasmid (Promega, USA) to construct the WT BCL2 reporter. BCL2 truncat plasmids and mutant plasmids were also constructed (Fig. 5E, H). For the Renilla luciferase reporter assay, each reporter construct was co-transfected into HEK293T cells together with the KLF5 plasmid or control plasmid. After 48 h of incubation, luciferase activity was measured using the Dual Luciferase Reporter Assay System (Promega, USA).

### Statistics

All statistical analyses were performed using SPSS version 20.0 software or R software 3.1.2 (R Core Team). Quantitative variables were analyzed using the Student’s *t*-test or ANOVA. Data are shown as mean ± SD. All experiments were performed in triplicates. Statistical significance was set at *P* < 0.05.

## Results

### Generation of patient-derived organoids from colorectal cancer

**Fig. 1:**
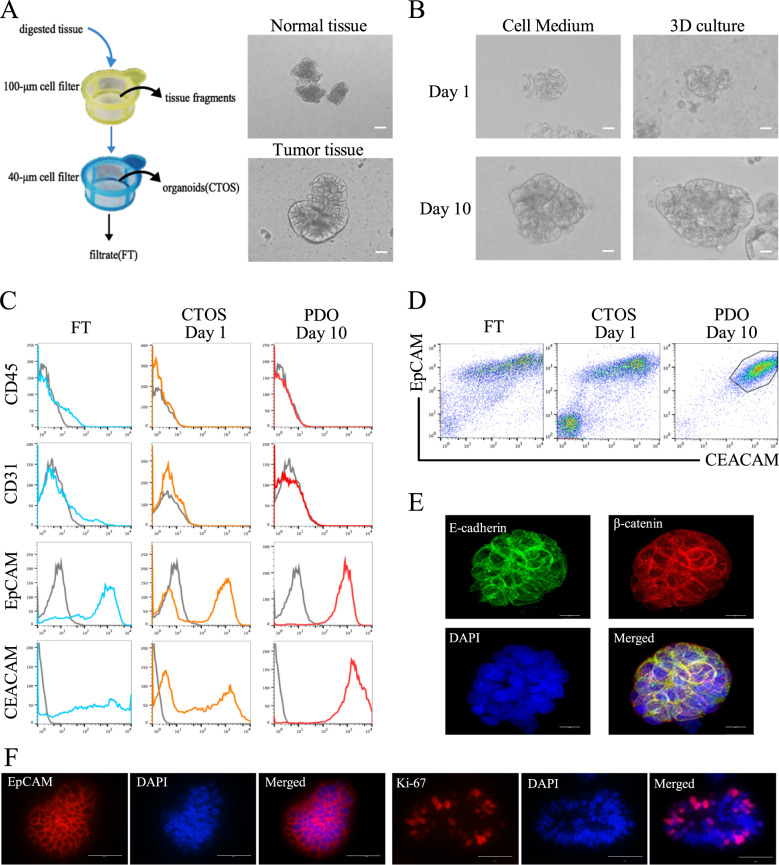
Generation of patient-derived organoids from colorectal cancer. **A** Schematic diagram for the preparation of CRC PDOs. Only tumor samples can form PDOs, and normal cells died in a short time. Scale, 100 μm. **B** The appearance of CRC PDO in suspension culture or 3D culture. Scale, 100 μm. **C**, **D** Flow cytometry showed that the CRC PDOs were highly purified cancer cell clusters. **E** E-cadherin (green) and β-catenin (red) staining of PDOs. E-cadherin was located on the cell membrane and linked to β-catenin. Scale, 100 μm. **F** PDOs expressed EpCAM and Ki-67. Scale, 200 μm. Data represent the mean ± SD.

CRC PDOs were constructed as described previously [7, 17]. We collected 20 tissue specimens from 14 CRC patients, including 14 tumor tissue specimens and 6 adjacent normal tissue specimens. All normal tissue samples (*n* = 6) were unable to form PDOs in our culture system (Fig. 1A). For the 14 tumor samples, one sample had bacterial infection and two samples failed due to too few tumor cells or a low proliferation rate (Table S1). Overall, we constructed a PDO culture success rate of approximately 78.5% (11 of 14 cases), which is consistent with previous reports [25]. PDOs were cultured in stem cell medium or in 3D Matrigel. The PDOs in 3D Matrigel grew slightly better than those in stem cell medium (Fig. S1B, C). PDOs could be mechanically divided and expanded to be continuously passaged for more than two months, providing enough cancer cells for subsequent experimental analysis.

### Characteristics of patient-derived CRC organoids

CRC PDOs were spherical and bright, with a smooth surface (Fig. 1A). The diameter of the PDOs was approximately 40–1,000 μm. A PDO with a diameter of 100 μm consisted of approximately 100 cells. CRC PDOs can proliferate stably in suspension cultures or 3D cultures (Fig. 1B). Since tumor tissue contains heterogeneous cellular components, we used flow cytometry to analyze the composition and purity of cells in CRC PDOs. The tested cells included the following three groups: group 1 was the flow-through (FT), which passed through the 40 μm filter when extracting PDOs; group 2 was the cell mass (CTOS Day 1), which remained in the 40 μm filter when extracting PDOs; and group 3 was the PDO after 10 days of culture (PDO Day 10). The results revealed that in addition to EpCAM+ epithelial cells, FT also contained CD31+ endothelial cells and CD45+ blood cells. In contrast, PDOs did not contain any CD45+ or CD31+ cells (Fig. 1C). In addition, when simultaneously labeling the expression of EpCAM and CEACAM in cells, it was found that compared with the PDO Day 1 group, the PDO Day 10 group only contained the EpCAM+/CEACAM+ cell population (Fig. 1D). The above results showed that the CRC PDOs constructed in vitro were highly purified cancer cell clusters. To identify whether PDOs originated or contained CSCs, flow cytometry analysis was used to detect the expression of CSC biomarkers CD1[26] and CD166 in PDOs. The results showed that PDOs contained CD133+/CD166+ cells, and the ratio of CD133+/CD166+ cells in PDOs increased with the extension of PDO culture time, indicating that PDOs contained CSCs (Fig. S1A). E-cadherin-mediated cell-to-cell contact is necessary to maintain the spatial structure of PDOs and cancer cell survival [27]. Therefore, we detected the expression of E-cadherin in PDOs. The results of immunofluorescence showed that E-cadherin expression was located at the cell membrane of all cells in PDOs and was linked by β-catenin (Fig. 1E). In addition, the immunofluorescence assay simultaneously characterized the expression of epithelial cell marker EpCAM and cell proliferation marker Ki-67 in PDOs (Fig. 1F).

### PDOs retained the characteristics of the original tumor

**Fig. 2:**
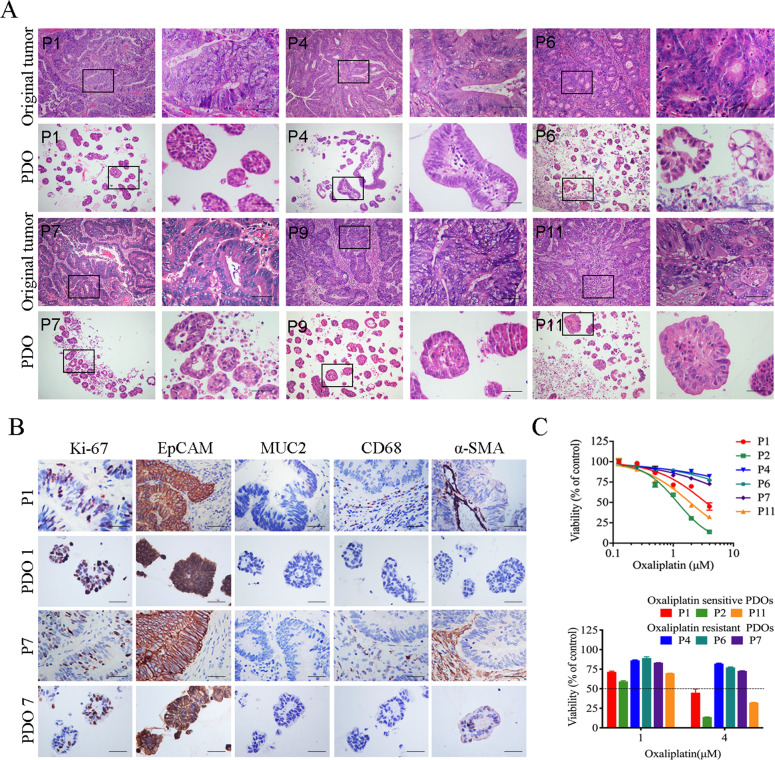
PDOs retained the characteristics of the original tumors. **A** H&E staining showed the internal structure of PDOs and original tumors. Scale, 200 μm. **B** The expression of Ki-67, EpCAM, MUC2, α-SMA, and CD68 in CRC PDOs and matched original tumors. Scale, 200 μm. **C** The dose-response curves of oxaliplatin. P1, P2, P11 were oxaliplatin-sensitive CRC PDOs. P4, P6 and P7 were oxaliplatin-resistant CRC PDOs. Data represent the mean ± SD.

CRC PDOs show histological features of adenocarcinoma, such as lumen structure and mucus production [7]. According to the differentiation level of the original tumor, CRC PDOs can show the histological features of highly differentiated adenocarcinoma cells with luminal structure, or of poorly differentiated adenocarcinoma cells without luminal structure (Fig. 2A). Immunohistochemical analysis was used to detect the expression of Ki-67, EpCAM, MUC2, α-SMA, and CD68 in CRC PDOs and matched original tumors. The expression levels of Ki-67 and EpCAM were retained in CRC PDOs, whereas MUC2, a differentiation marker, was not expressed in either the original tumor or PDOs (Fig. S1D). In contrast, α-SMA, a marker of activated fibroblasts, and CD68, a macrophage marker, were detected in the microenvironment of the original tumor, but not in the PDOs (Fig. 2B). These results are consistent with those of previous reports [28].

### Oxaliplatin sensitivity assay and drug screening assay with CRC PDOs

We previously verified that CRC PDOs are composed of highly purified tumorigenic cells and retain the characteristics of parental tumors. Therefore, PDOs may be used for chemosensitivity assays in vitro. Oxaliplatin, a key drug widely used in clinical chemotherapy for CRC, was chosen to conduct drug sensitivity assays of PDOs from 12 CRC patients (Table S10). All PDOs were exposed to oxaliplatin and at indicated concentrations, and were calculated the growth ratio of the PDOs after drug exposure and fitted the dose-response curves. 12 PDOs were divided into three groups (sensitive, moderate, and resistant) by calculating the AUC value (Fig. S1E, F). The results of drug sensitivity experiments in vitro were consistent with clinical data, suggesting the potential of PDOs to detect the chemical sensitivity of colorectal cancer (Fig. S1G). The IC50 value of 4 μM was chosen as the cut-off value between the oxaliplatin-sensitive vs. resistant models according to the results of oxaliplatin sensitivity assays. For example, in the PDOs constructed from P1, the growth of PDOs was significantly inhibited at 4 μM (Fig. S2C); in the PDOs constructed from P2, the growth of PDOs was significantly inhibited at 2 μM (Fig. S2D); and in PDOs constructed from P4, even 4 μM oxaliplatin was not enough to inhibit the growth of PDOs at the same level (Fig. S2E). We have confirmed that PDOs constructed from different patients have significant differences in response to oxaliplatin treatment, but the underlying mechanism remains unclear. Therefore, we selected oxaliplatin-resistant PDOs (P4, P6, and P7) for further research (Fig. 2C). Sixty FDA-approved anti-tumor drugs and small molecule compounds were used to conduct drug screening assays, and the results showed that compared with monotherapy, the KLF5 inhibitor ML264 significantly increased oxaliplatin sensitivity in oxaliplatin-resistant PDOs (Fig. S3A).

### ML264 restores oxaliplatin sensitivity in CRC PDOs by restoring the apoptotic response

**Fig. 3:**
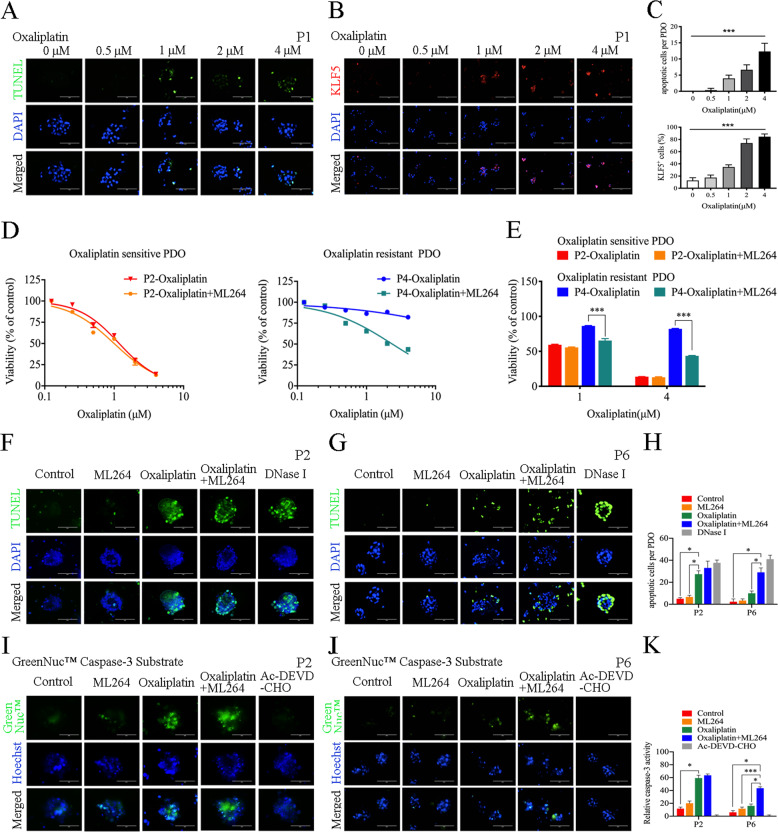
ML264 restored oxaliplatin sensitivity in CRC PDOs by restoring the apoptotic response. **A** Images of TUNEL (green) apoptosis detection in PDOs treated with oxaliplatin. Scale, 200 μm. **B** KLF5 (red) staining of PDOs treated with oxaliplatin at indicated concentrations. Scale, 200 μm. **C** Oxaliplatin significantly induced apoptosis in a dose-dependent manner. The proportion of KLF5+ cells significantly increased as the dose of oxaliplatin treatment increased (mean ± SD, *n* = 3 for each group, one-way ANOVA, *** *p* < 0.001). **D**, **E** The dose-response curves of combined treatment of ML264 and oxaliplatin. ML264 reduced the dose of oxaliplatin required to inhibit the growth of oxaliplatin-resistant CRC PDO. **F**, **G** Images of TUNEL (green) apoptosis detection in P2 and P6 treated as indicated. DNase I was used as a positive control. Scale, 200 μm. **H** ML264 restored oxaliplatin-induced apoptotic response in oxaliplatin-resistant CRC PDO (mean ± SD, *n* = 3 for each group, one-way ANOVA, *P < 0.05, **P < 0.01, *** *p* < 0.001). **I**, **J** The activity of caspase 3 (green) detection of P2 and P6. Ac-DEVD-CHO was used as a negative control. Scale, 200 μm. **K** ML264 restored oxaliplatin-induced activity of caspase 3 in oxaliplatin-resistant CRC PDO (mean ± SD, *n* = 3 for each group, one-way ANOVA, *P < 0.05, *** *p* < 0.001). The concentration of ML264 was 10 μM, and the concentration of oxaliplatin was 2 μM.

To further explore the underlying mechanism by which ML264 enhances the sensitivity of CRC PDOs to oxaliplatin, we tested the apoptosis level and the expression of KLF5 in PDOs. The TUNEL Apoptosis Detection Kit was used to detect the level of apoptosis in PDOs. In oxaliplatin-sensitive PDOs constructed from P1, oxaliplatin significantly induced apoptosis in a dose-dependent manner (Fig. 3A, C), and the proportion of KLF5+ cells significantly increased as the dose of oxaliplatin treatment increased (Fig. 3B, C). On the contrary, in oxaliplatin-resistant PDOs constructed from P4, high concentrations of oxaliplatin still could not induce significantly high levels of apoptosis (Fig. S4A, C). The proportion of of KLF5+ cells was high in untreated oxaliplatin-resistant PDOs, while KLF5 expression could be up-regulated after high concentration of oxaliplatin treatment (Fig. S4B, C). These data indicate that KLF5 may inhibit in the apoptosis process induced by oxaliplatin, and ML264 may enhance the sensitivity to oxaliplatin by inhibiting the function of KLF5. Next, we verified whether ML264 could restore oxaliplatin sensitivity and oxaliplatin-related apoptosis in CRC PDOs. It was found that ML264 significantly reduced the viability of oxaliplatin-resistant PDO (P4) upon oxaliplatin treatment, and had no significant effect on oxaliplatin-sensitive PDO (P2) (Fig. 3D, E). Analysis of the apoptosis level of CRC PDOs showed that ML264 significantly restored the apoptosis of oxaliplatin-resistant PDO (P4 and P6), and had no significant effect on oxaliplatin-sensitive PDO (P2) (Fig. 3F–H, Fig. S4D–F). In addition, the activity of caspase 3 was also significantly increased in oxaliplatin-resistant PDO (P6) but not in oxaliplatin-sensitive PDO (P2) when treated with oxaliplatin combined with ML264 (Fig. 3I, J, K). These results indicate that the KLF5 inhibitor ML264 can restore the apoptotic response and enhance the oxaliplatin sensitivity of CRC PDOs.

### The KLF5/Bcl-2/caspase3 signal pathway affects oxaliplatin-induced apoptosis of CRC cells

**Fig. 4:**
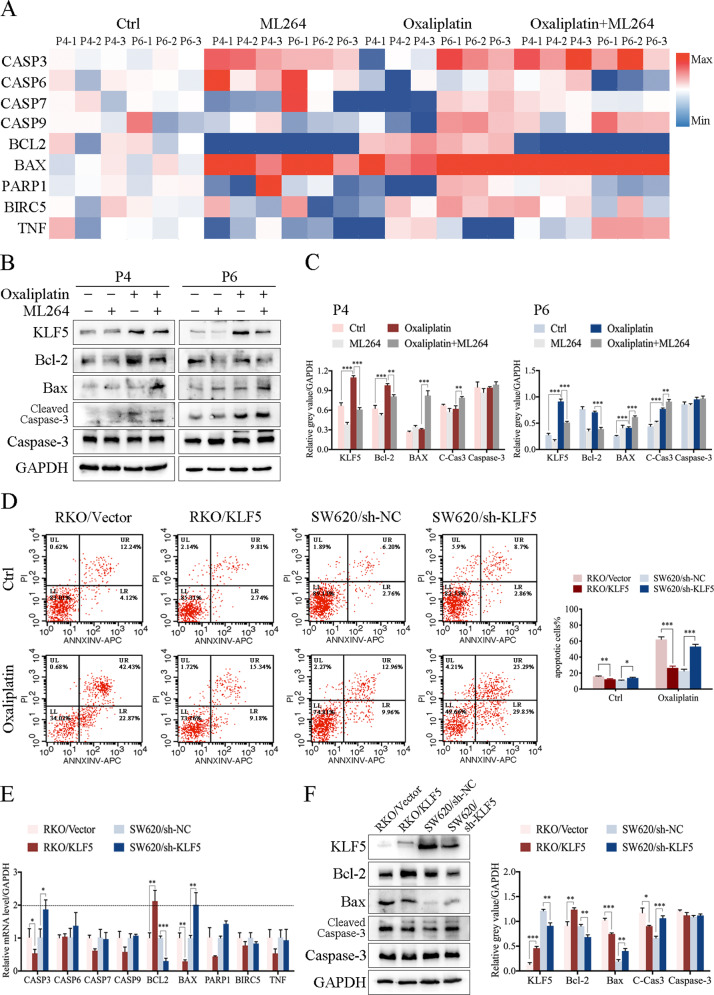
Oxaliplatin induced the expression of KLF5 and a variety of apoptosis-related proteins. **A** qPCR showed the expression of apoptosis-related genes of PDOs treated with oxaliplatin with or without ML264. **B** Western blotting of KLF5, Bcl-2, Bax, cleaved caspase 3 and caspase 3 in PDOs. **C** Oxaliplatin induced the expression of KLF5, Bcl-2 and Bax. Oxaliplatin combined with ML264 induced the expression of Bax and cleaved caspase 3, and inhibited the expression of KLF5 and Bcl-2 (mean ± SD, *n* = 3 for each group, one-way ANOVA, **P < 0.01, *** *p* < 0.001). **D** Apoptosis detection showed that KLF5 promoted apoptosis in CRC cell lines with or without oxaliplatin treated (mean ± SD, *n* = 3 for each group, Student’s *t*-test, *P < 0.05, **P < 0.01, *** *p* < 0.001). **E** The expression of apoptosis-related genes of CRC cell lines (mean ± SD, *n* = 3 for each group, Student’s *t*-test, *P < 0.05, **P < 0.01, *** *p* < 0.001). **F** Western blotting of KLF5, Bcl-2, Bax, cleaved caspase 3 and caspase 3 in CRC cell lines. KLF5 promoted the expression of Bcl-2 and inhibited the expression of Bax and cleaved caspase 3 (mean ± SD, *n* = 3 for each group, Student’s *t*-test, *P < 0.05, **P < 0.01, *** *p* < 0.001). The concentration of ML264 was 10 μM, and the concentration of oxaliplatin was 2 μM.

Previous studies have reported many molecules involved in drug-induced apoptosis, including the caspase family, Bcl family, PARP1, BIRC5, and TNF [29–33]. To further investigate the underlying molecular mechanism, oxaliplatin-resistant PDOs were divided into four groups (Group 1: Control; Group 2: ML264 10 μM; Group 3: oxaliplatin 2 μM; Group 4: oxaliplatin 2 μM + ML264 10 μM). Then, qPCR and western blotting were used to detect the expression of apoptosis-related molecules. The results of qPCR showed that at the RNA level, oxaliplatin significantly induced the expression of Bcl-2 and Bax, whereas in the oxaliplatin + ML264 treated group, the expression of Bcl-2 was inhibited, while the expression of Bax and CASP-3 increased (Fig. 4A). Similarly, western blotting revealed that after treatment with oxaliplatin combined with ML264, the expression of the anti-apoptotic molecule Bcl-2 decreased, while the expression of pro-apoptotic Bax and the cleavage of caspase-3 increased (Fig. 4B, C).

Next, CRC cell lines were used to further explore the downstream molecular signaling pathways of KLF5. According to the results of western blotting of CRC cell lines, KLF5 was relatively highly expressed in SW620 cells and low in RKO cells (Fig. S3B). We generated stably transfected CRC cell lines, including RKO control (RKO/Vector), RKO with KLF5 overexpression (RKO/KLF5), SW620 control (SW620/sh-NC), and SW620 with KLF5 downregulation (SW620/sh-KLF5). Next, we detected the sensitivity counterparts to oxaliplatin of parental cells vs. their transfected cells, and the IC-50 values for RKO/Vector, RKO/KLF5, SW620/sh-NC and SW620/sh-KLF5 were 2.931, 5.002, 5.411 and 3.290 for 72 h (Fig. S3C). To evaluate the role of KLF5 in oxaliplatin-induced apoptosis in CRC cells, flow cytometry was used to detect the level of apoptosis with or without oxaliplatin treatment. Compared with the control group, RKO/KLF5 significantly suppressed cell apoptosis, while SW620/sh-KLF5 significantly promoted cell apoptosis. When treated with 2 μM oxaliplatin, the anti-apoptotic effect mediated by KLF5 was significantly enhanced in CRC cells (Fig. 4D). In addition, it was found that ML264 significantly increased apoptosis upon oxaliplatin treatment in SW620/sh-NC and RKO/KLF5, and had no significant effect on SW620/sh-KLF5 and RKO/Vector (Fig. S3D). Similarly, we used qPCR and western blotting to detect the expression of apoptosis-related molecules in stably transfected CRC cell lines. The results of qPCR showed that KLF5 significantly induced the expression of Bcl-2 but inhibited the expression of Bax and CASP3 (Fig. 4E). At the protein level, KLF5 also promoted the expression of Bcl-2 and inhibited the expression of Bax (Fig. 4F). Although KLF5 cannot regulate the expression of Caspase-3 at the protein level, it significantly suppresses the cleavage of caspase-3, which directly acts as an effector of caspase 3 to participate in the apoptotic response [34].

**Fig. 5:**
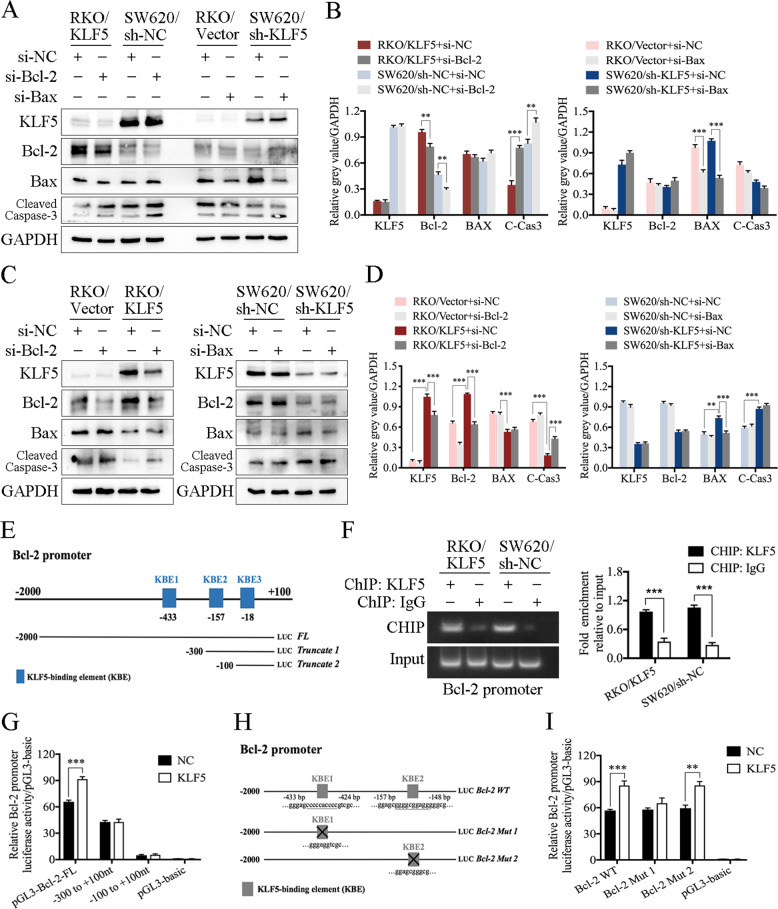
The KLF5/Bcl-2/caspase 3 signaling pathway affects the oxaliplatin-induced apoptosis of CRC cells. **A** Western blotting of KLF5, Bcl-2, Bax, cleaved caspase 3 in CRC cell lines. **B** Knocking down Bcl-2 significantly promoted the expression of cleaved caspase 3, whereas knocking down Bax did not affect the expression of Bcl-2 or cleaved caspase 3 (mean ± SD, *n* = 3 for each group, Student’s *t*-test, **P < 0.01, *** *p* < 0.001). **C**, **D** Western blotting showed that inhibiting Bcl-2 can reverse the function of KLF5 in suppressing the expression of cleaved caspase 3, whereas inhibiting Bax cannot reverse the function of KLF5 in cleaved caspase 3 suppression (mean ± SD, *n* = 3 for each group, one-way ANOVA, **P < 0.01, *** *p* < 0.001). **E** KLF5-binding elements (KBE1 to KBE3) on Bcl-2 promoter region. **F** ChIP verified the direct binding of KLF5 to the predicted site (KBE1) of the Bcl-2 promoter (mean ± SD, *n* = 3 for each group, Student’s *t*-test, *** *p* < 0.001). **G** The results of luciferase reporter assay showed that KLF5 only promoted the luciferase activity of pGL3-Bcl2-FL (mean ± SD, *n* = 3 for each group, Student’s *t*-test, *** *p* < 0.001). **H** Schematic diagram showed the mutation site of Bcl-2 Mut 1 and Bcl-2 Mut 2 plasmids. **I** The results of luciferase reporter assay showed that KLF5 could not promote the luciferase activity of Bcl-2 Mut 1, which did not contain KBE1 sequence (mean ± SD, *n* = 3 for each group, Student’s *t*-test, **P < 0.01, *** *p* < 0.001).

Small interfering RNA technology was used to knockdown Bcl-2 and Bax in CRC cells to investigate the pathways mediating KLF5-related cell apoptosis. We confirmed that KLF5 significantly induced the expression of Bcl-2, so we first knocked down Bcl-2 in KLF5 highly expressing cells (RKO/KLF5 and SW620/sh-NC). The results showed that Bcl-2 knockdown did not affect the expression of Bax, but significantly promoted the cleavage of caspase-3. Similarly, according to our previous results, KLF5 suppressed Bax expression. Next, we chose KLF5 weakly expressing cells (RKO/Vector and SW620/sh-KLF5) to knockdown Bax and found that knocking down Bax did not affect the expression of Bcl-2 or the cleavage of caspase-3 (Fig. 5A, B). We further confirmed whether Bcl-2 was the main executor in KLF5 mediating anti-apoptosis response. The results of western blotting showed that inhibiting the expression of Bcl-2 can reverse the function of KLF5 to inhibit apoptosis. In contrast, inhibiting the expression of Bax did not reverse the anti-apoptotic effect of KLF5 (Fig. 5C, D). These results suggest that the inhibitory effect of KLF5 on cell apoptosis is mainly mediated by Bcl-2 and its downstream molecule caspase 3.

### KLF5 promotes the transcription of Bcl-2 in CRC cells

Because KLF5 can directly bind to the promoter region of target genes and regulate the transcription and expression of downstream genes [35], we further explored the mechanism of KLF5-induced Bcl-2 gene transcription. We first predicted three KLF5-binding elements (KBE1 to KBE3) in the Bcl-2 promoter region using JASPAR software (Fig. 5E). Then, the plasmids of the full-length Bcl-2 promoter region (pGL3-Bcl2-FL) and two truncated Bcl-2 promoter fragments (truncated 1: −300 to +100 nt, truncated 2: −100 to +100 nt) were constructed (Fig. 5E). pGL3-basic was used as a negative control. A luciferase reporter assay was used to detect the transcriptional regulation of KLF5 on the constructed luciferase plasmid. The results showed that overexpression of KLF5 promoted the luciferase activity of pGL3-Bcl2-FL. In contrast, KLF5 overexpression had no significant effect on luciferase activity in truncated 1 and truncated 2 plasmids, which did not contain the KBE1 sequence (Fig. 5G). In addition, oxaliplatin treatment promoted the luciferase activity of pGL3-Bcl2-FL (Fig. S4G). The sequences of KBE1 and KBE2 were deleted from the full-length Bcl-2 promoter plasmid (Bcl-2 WT) to construct Bcl-2 Mut 1 and Bcl-2 Mut 2 plasmids (Fig. 5H). As expected, the KBE1 mutation significantly reduced the activation of the Bcl-2 promoter (Fig. 5I). Subsequently, we designed a PCR primer for the Bcl-2 promoter region based on the results of the luciferase reporter assay. ChIP verified the direct binding of KLF5 to the predicted site (KBE1) of the Bcl-2 promoter (Fig. 5F). These results suggested that the effective KLF5-binding site on the Bcl2 promoter was between −433 and −424 nt (KBE1).

### Inhibition of KLF5 overcomes oxaliplatin resistance in xenograft tumors

**Fig. 6:**
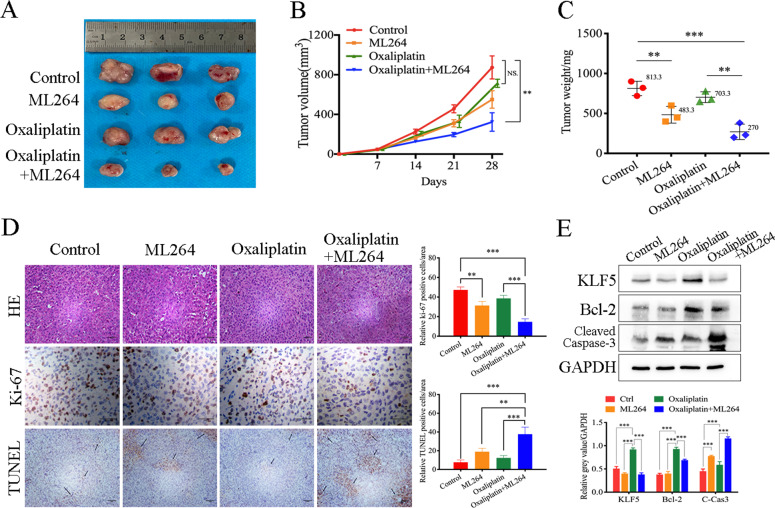
Inhibition of KLF5 overcomes oxaliplatin resistance in xenograft tumors. **A**, **B** Nude mice were injected s.c. with CRC cells. After tumor diameter reached approximately 5 mm, mice were treated with oxaliplatin alone or in combination with ML264 as indicated. Tumor volume at indicated time points was calculated and plotted (mean ± SD, *n* = 3 for each group, one-way ANOVA, **P < 0.01, NS not significant). **C** The combined use of ML264 can significantly inhibit tumor growth in xenograft tumors (mean ± SD, *n* = 3 for each group, one-way ANOVA, **P < 0.01, *** *p* < 0.001). **D** TUNEL and Ki-67 immunostaining were used to analyze the apoptosis level of xenograft tumors. Arrows indicate example cells with positive staining. ML264 significantly restored the oxaliplatin-induced tumor apoptosis (mean ± SD, *n* = 3 for each group, one-way ANOVA, **P < 0.01, *** *p* < 0.001). Scale, 200 μm. **E** Western blotting was used to analysis KLF5, Bcl-2 and cleaved caspase 3. The concentration of ML264 was 25 mg/kg, and the concentration of oxaliplatin was 5 mg/kg (mean ± SD, *n* = 3 for each group, one-way ANOVA, **P < 0.01, *** *p* < 0.001).

We compared the effects of oxaliplatin or oxaliplatin combined with ML264 on constructed CRC xenograft tumors in nude mice to determine whether KLF5 inhibition can restore oxaliplatin sensitivity in vivo. The results showed that the combined use of ML264 significantly inhibited tumor growth in xenograft tumors (Fig. 6A, B, C). In addition, although the mice had tumor-related weight loss, the drug treatment had no significant effect on the weight of the mice (Fig. S3E). TUNEL and Ki-67 immunostaining showed that compared with the control and oxaliplatin single-treatment groups, ML264 significantly restored oxaliplatin-induced tumor apoptosis (Fig. 6D). Western blotting showed that oxaliplatin treatment significantly promoted the expression of KLF5 and Bcl-2 in xenograft tumors. However, the combined treatment of ML264 and oxaliplatin inhibited the expression of KLF5 and Bcl-2 and promoted the cleavage of caspase-3 (Fig. 6E).

## Discussion

In the clinical treatment of colorectal cancer, systemic treatment (neoadjuvant or adjuvant cytotoxic chemotherapy) is often used in combination with surgical treatment [36]. However, for many CRC patients, this method only provides a moderate benefit in terms of survival [37]. Therefore, there is an urgent need to develop more effective treatment methods to solve the problem of drug resistance. In the development of novel anticancer therapies, predictive in vitro models are highly needed to help match patients to treatments. Chemotherapy and targeted drugs can be first used in these models to test their safety and effectiveness instead of being used directly in patients [38]. PDOs can be effectively constructed from patient tumor tissues and have a faithful response and maintain the characteristics of parental tumors, with individual differences. Moreover, tumor PDOs can reliably reflect the response of corresponding patients to the same drug [39]. We identified CRC organoids that were constructed based on the characteristics of PDOs. The results showed that CRC PDOs were composed of highly purified colorectal cancer cells without other cellular components. In addition, CRC PDOs retained the characteristics of the original tumor, including similarities in histology and consistency in protein expression. We further tested the ability of PDOs to mimic the response to chemotherapy in patients. The oxaliplatin sensitivity assay showed that the response of PDOs to oxaliplatin had individual differences, suggesting the potential of PDOs to detect individual chemical sensitivity of colorectal cancer.

In addition to the personalized testing of chemotherapy and targeted therapy, PDOs are also suitable for high-throughput drug screening [40]. In particular, PDOs can test various drug combinations in vitro to generate new treatments, which may help us understand the potential molecular mechanisms of tumor resistance [26]. We used oxaliplatin-resistant CRC PDOs to conduct drug screening assays and tested the effect of 60 FDA-approved anti-tumor drugs and small molecule compounds in combination therapy with oxaliplatin in colorectal cancer. The results showed that ML264 can restore oxaliplatin sensitivity in CRC PDOs, indicating that inhibition of KLF5 is an effective approach to resensitize CRC with oxaliplatin resistance. The resistance of malignant tumors to chemotherapy is mostly mediated by anti-apoptotic effects [41]. Therefore, we suspected that KLF5 is involved in oxaliplatin-induced apoptosis in colorectal cancer. The results of apoptosis detection showed that inhibition of KLF5 can restore the apoptotic response of CRC cells and enhance oxaliplatin sensitivity in CRC PDOs. Similarly, treatment of CRC xenograft tumors demonstrated that inhibition of KLF5 can overcome oxaliplatin resistance in vivo. Previous studies have reported many molecules involved in drug-induced apoptosis [42]. In our study, we confirmed that ML264 affected oxaliplatin-induced CRC apoptosis by inhibiting the Bcl-2/caspase3 signaling pathway. The results of the luciferase reporter assay and ChIP showed that KLF5 promoted the expression of Bcl-2 through transcriptional regulation, thereby exerting its anti-apoptotic function.

In summary, our study provides reliable laboratory evidence that KLF5 is a promising therapeutic target for CRC, especially CRC resistant to oxaliplatin. We further provide new evidence for the application of PDO models in anti-tumor drug resistance studies. That is, PDOs can be used for drug screening, and further research can be conducted based on the target of effective drugs. However, this study also has certain limitations. Relying too much on the examination results of tumor organoids also poses certain risks. A large number of biobanking studies have confirmed that PDOs faithfully capture the genomic features of the primary tumor and much of the genomic diversity of CRC [7]. It is not clear whether PDOs can reflect intratumoral heterogeneity, which may cause heterogeneity in the response of PDOs to the drug and interfere with the researcher’s judgment [42]. Therefore, more clinical trials are needed to measure the sensitivity and specificity of PDOs in patients receiving drug treatment. In conclusion, tumor organoids can fill the gap between cancer research and clinical trials and act as a promising tool model for future scientific research and clinical treatment.

## Conclusions

**Fig. 7:**
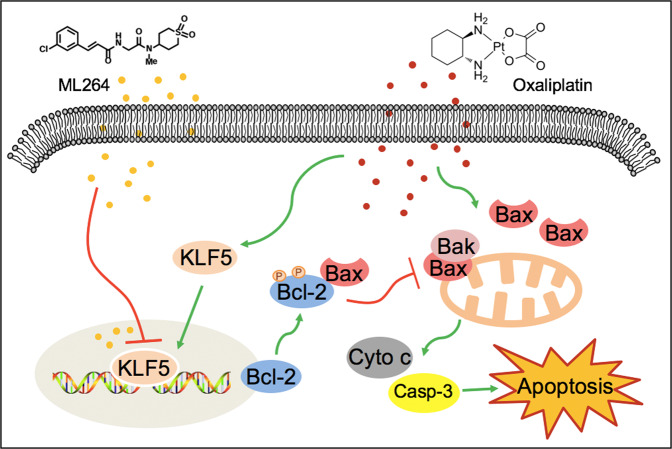
Schematic diagram shows the mechanisms of how the CRC cells’ resistance to oxaliplatin and ML264 restores oxaliplatin sensitivity of colorectal cancer. Overexpression of KLF5 and its downstream anti-apoptotic factor Bcl-2 was one of the mechanisms for CRC to resist oxaliplatin.

This study indicated that the overexpression of KLF5 and its downstream anti-apoptotic factor Bcl-2 is one of the mechanisms by which CRC resists oxaliplatin. The combined treatment of ML264 and oxaliplatin reversed oxaliplatin resistance in CRC and promoted the apoptosis of tumor cells (Fig. 7).

## Supplementary information

Supplementary figure 1

Supplementary figure 2

Supplementary figure 3

Supplementary figure 4

Supplementary figure legends

Supplementary tables

aj-checklist

Original Data File

S1-Targetmol Anti-cancer Compound Library-3338cpds

S2-Targetmol Anti-cancer Compound Library-134cpds

S3-A collection of 60 Custom Compounds.

## Data Availability

The datasets supporting the conclusions of this article are included within the article and its additional files.
