# Supplementary figures and images for: KLF5 inhibition overcomes oxaliplatin resistance in patient-derived colorectal cancer organoids by restoring apoptotic response
Source: Cell Death Dis. 2022 Apr 5;13(4):303. doi: 10.1038/s41419-022-04773-1 (PMC8980070; doi:10.1038/s41419-022-04773-1)

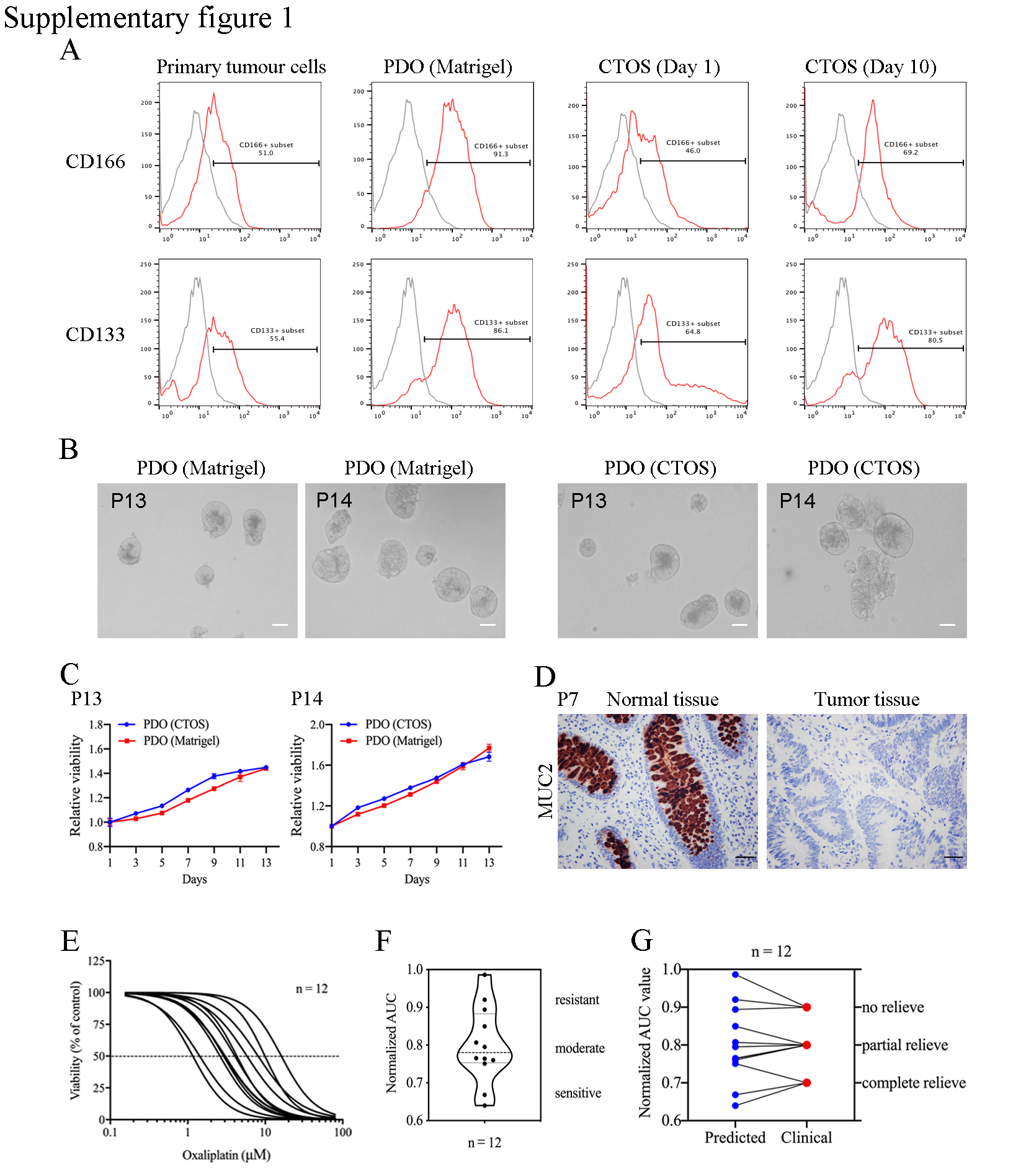

Supplement: Supplementary file 1 — Supplementary figure 1 [file 41419_2022_4773_MOESM1_ESM.tif]

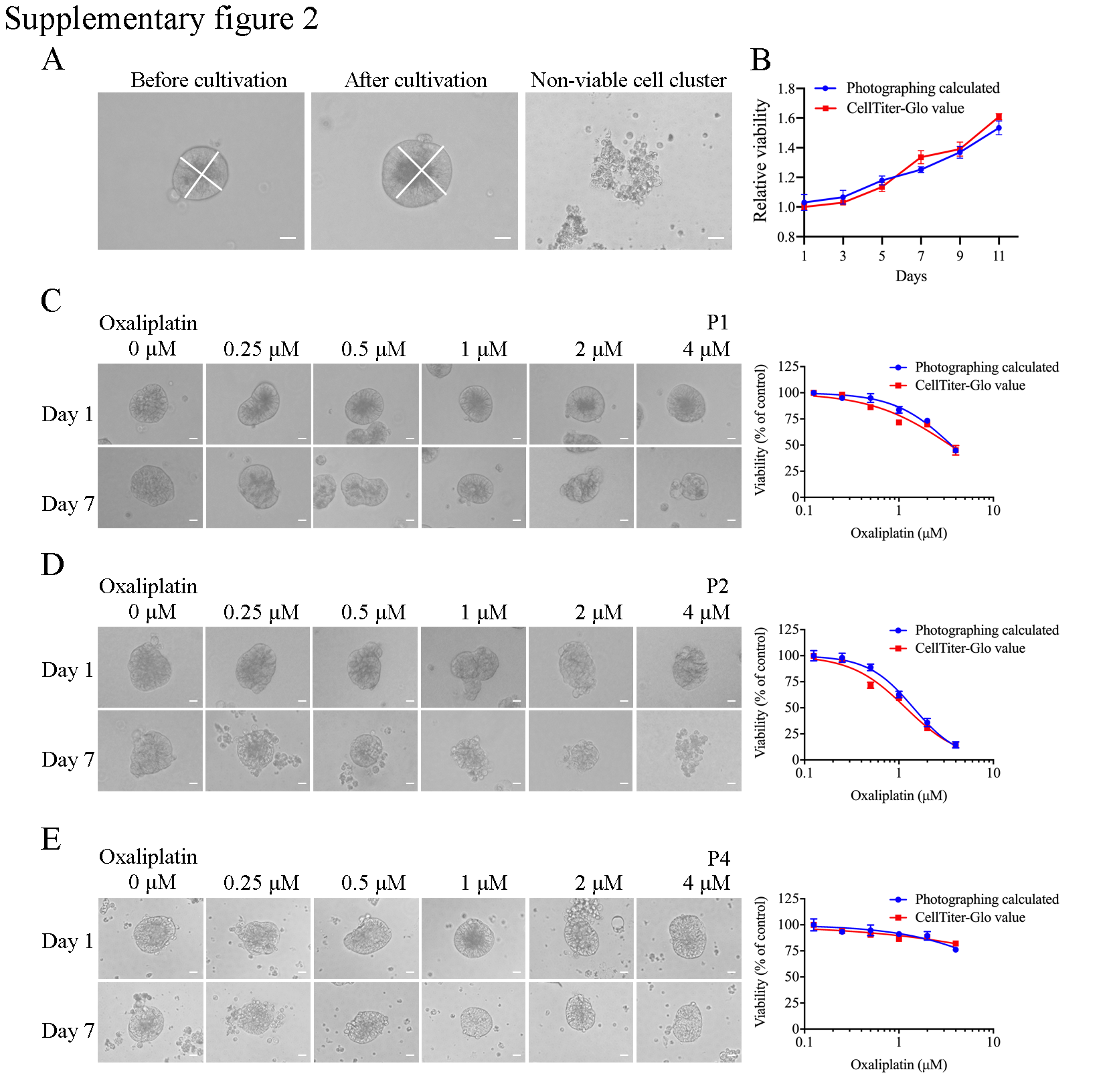

Supplement: Supplementary file 2 — Supplementary figure 2 [file 41419_2022_4773_MOESM2_ESM.tif]

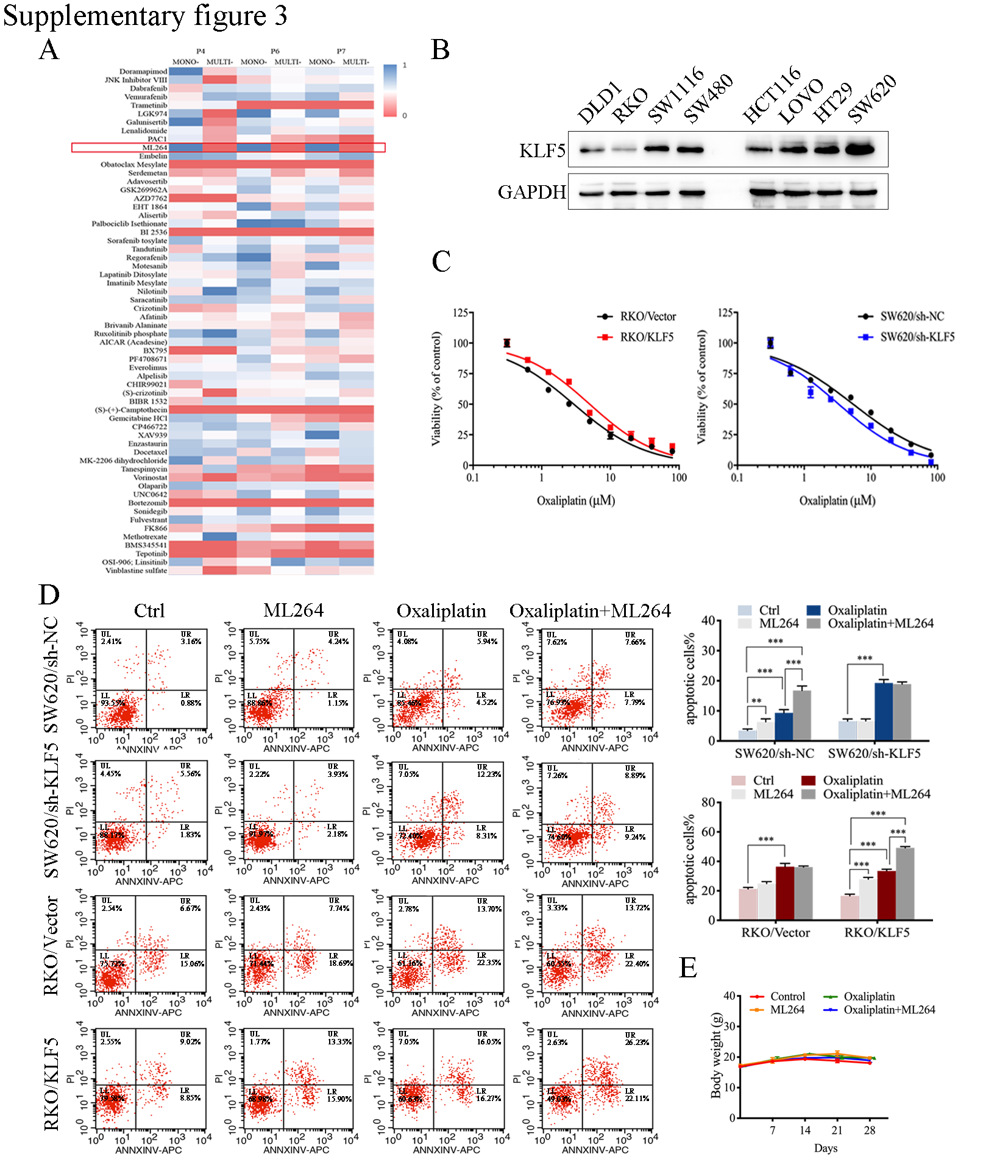

Supplement: Supplementary file 3 — Supplementary figure 3 [file 41419_2022_4773_MOESM3_ESM.tif]

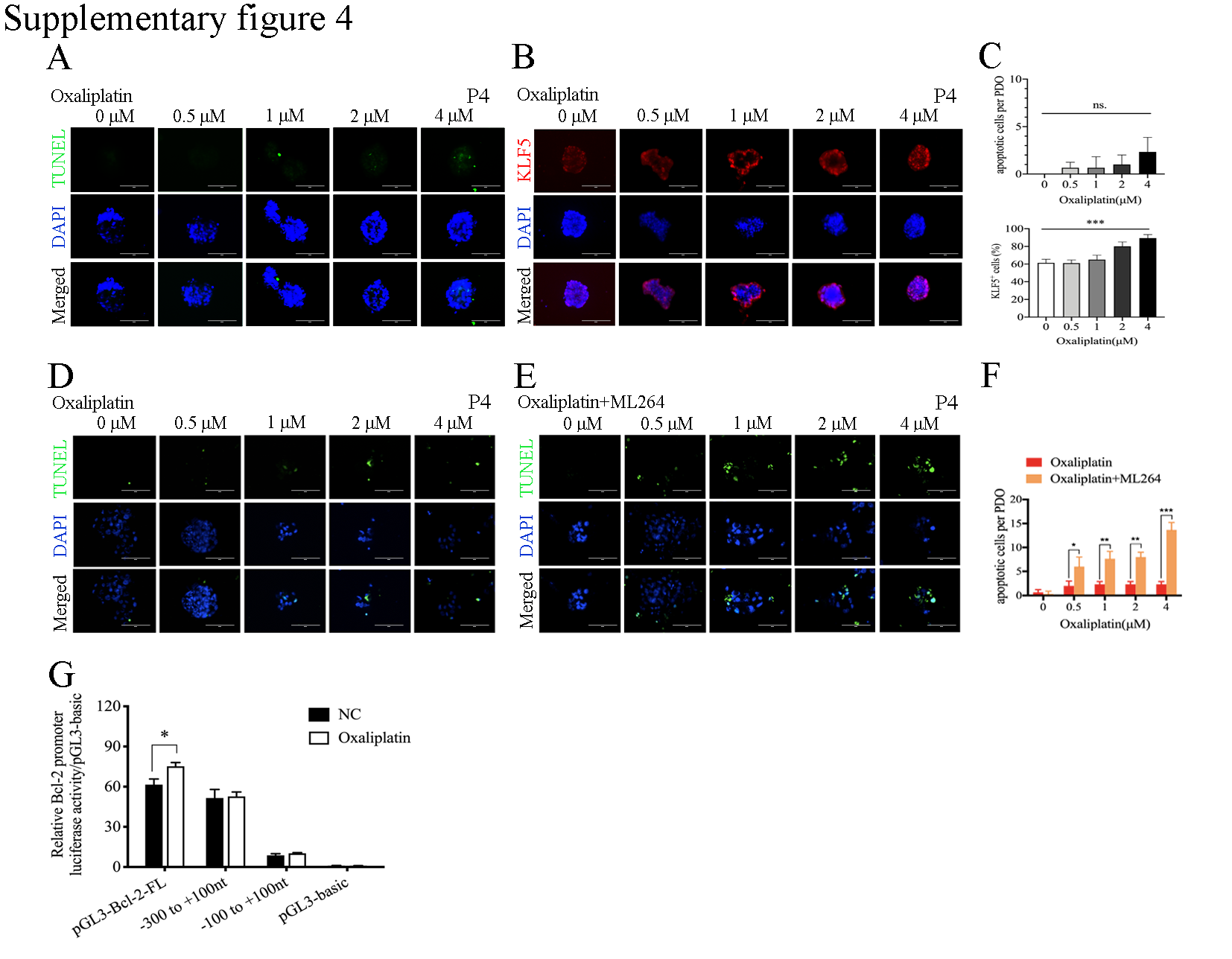

Supplement: Supplementary file 4 — Supplementary figure 4 [file 41419_2022_4773_MOESM4_ESM.tif]
