# Supplementary material for: KLF5 inhibition overcomes oxaliplatin resistance in patient-derived colorectal cancer organoids by restoring apoptotic response
Source: Cell Death Dis. 2022 Apr 5;13(4):303. doi: 10.1038/s41419-022-04773-1 (PMC8980070; doi:10.1038/s41419-022-04773-1)
Supplement: Supplementary file 5 — Supplementary figure legends [file 41419_2022_4773_MOESM5_ESM.docx]

**Supplementary figure legends**

**Supplementary figure 1** PDOs retained the characteristics of the original tumors. (A) Flow cytometry showed the expression of CD133 and CD166 in PDOs. PDOs contained CD133+/CD166+ cells, and the ratio of CD133+/CD166+ cells in PDOs increased with the extension of PDO culture time. (B) The appearance of CRC PDO constructed by CTOS or Matrigel methods. Scale, 100 μm. (C) The growth state of PDOs constructed by CTOS or Matrigel methods. (D) The expression of MUC2 in normal tissue or CRC tissue. Scale, 200 μm. (E) The dose-response curves of oxaliplatin in 12 CRC PDOs. (F) CRC PDOs were divided into three groups (sensitive, moderate, and resistant) by calculating the AUC value. (G) The results of drug sensitivity experiments *in vitro* were consistent with clinical data. Data represent the mean ± SD.

**Supplementary figure 2** The results of oxaliplatin sensitivity assays of CRC PDOs. (A) Schematic diagram for the calculation of individual cells in 3D structures. Scale, 100 μm. (B) The viability of the organoids was measured by the CellTiter-Glo® luminescent cell viability assay and photographing calculation. The two evaluation methods were consistent with the counting results for the same sample. (C) The results of oxaliplatin sensitivity assays of P1, which was moderately sensitive to oxaliplatin. Scale, 100 μm. (D) The results of oxaliplatin sensitivity assays of P2, which was highly sensitive to oxaliplatin. Scale, 100 μm. (E) The results of oxaliplatin sensitivity assays of P4, which was resistant to oxaliplatin. Scale, 100 μm. Data represent the mean ± SD.

**Supplementary figure 3**

(A) The results of drug screening assay, and ML264 combined with oxaliplatin significantly improved the treatment effect of oxaliplatin-resistant PDOs compared with monotherapy. The concentration of the compounds used was 3 μM. (B) Western blotting of KLF5 in CRC cell lines. (C) The dose-response curves of oxaliplatin of RKO/Vector vs. RKO/KLF5 and SW620/sh-NC vs. SW620/sh-KLF5. (D) Apoptosis detection showed that ML264 increased apoptosis upon oxaliplatin treatment in SW620/sh-NC and RKO/KLF5 (mean ± SD, n = 3 for each group, one-way ANOVA, **P < 0.01, ***, p < 0.001). (E) The weight data of nude mice. The concentration of ML264 was 10 μM, and the concentration of oxaliplatin was 2 μM. Data represent the mean ± SD. *P < 0.05, **P < 0.01, ***P < 0.001.

**Supplementary figure 4**

(A) Images of TUNEL (green) apoptosis detection in PDOs treated with oxaliplatin. Scale, 200 μm. (B) KLF5 (red) staining of PDOs treated with oxaliplatin at indicated concentrations. Scale, 200 μm. (C) Oxaliplatin did not induced apoptosis in oxaliplatin-resistant PDOs. The proportion of KLF5+ cells significantly increased as the dose of oxaliplatin treatment increased (mean ± SD, n = 3 for each group, one-way ANOVA, ***, p < 0.001). (D&E&F) Images of TUNEL (green) apoptosis detection in PDOs treated with oxaliplatin and ML264. ML264 restored oxaliplatin-induced apoptotic response in oxaliplatin-resistant PDOs (mean ± SD, n = 3 for each group, Student's t-test, ***, p < 0.001).. Scale, 200 μm. (G) The results of luciferase reporter assay showed that oxaliplatin promoted the luciferase activity of pGL3-Bcl2-FL (mean ± SD, n = 3 for each group, Student's t-test, ***, p < 0.001).
