# Supplementary material for: KLF5 inhibition overcomes oxaliplatin resistance in patient-derived colorectal cancer organoids by restoring apoptotic response
Source: Cell Death Dis. 2022 Apr 5;13(4):303. doi: 10.1038/s41419-022-04773-1 (PMC8980070; doi:10.1038/s41419-022-04773-1)
Supplement: Supplementary file 6 — Supplementary tables [file 41419_2022_4773_MOESM6_ESM.docx]

**Table S1 Clinical characteristics of PDOs donor.**

| Sample ID | Patient age | Patient sex | Tumor location | TNM stage | PDOs | | |
| --- | --- | --- | --- | --- | --- | --- | --- |
|  |  |  |  |  | Formation | Growth | Drug |
| P1 | 59 | M | A | IIA | Yes | Yes | Yes |
| P2 | 76 | F | R | IIIB | Yes | Yes | Yes |
| P3 | 83 | M | R | IIA | No | No | No |
| P4 | 57 | M | S | IV | Yes | Yes | Yes |
| P5 | 33 | M | S | IV | Yes | Yes | No |
| P6 | 57 | M | T | IV | Yes | Yes | Yes |
| P7 | 54 | M | S | IIIB | Yes | Yes | Yes |
| P8 | 66 | M | R | IIIB | Yes | No | No |
| P9 | 86 | M | A | IIA | Yes | Yes | No |
| P10 | 72 | M | D | IIA | Yes | Yes | No |
| P11 | 73 | M | R | IIIB | Yes | Yes | Yes |
| P12 | 72 | M | S | IIIB | Yes | No | No |
| P13 | 54 | M | S | IIIB | Yes | Yes | No |
| P14 | 73 | F | T | IIIB | Yes | Yes | No |

Tumor location: A, ascending colon; T, transverse colon; D, descending colon; S, sigmoid colon, R, rectum.

**Table S2 Reagents information**

| **Reagent** | **Catalog** | **Manufacturer** |
| --- | --- | --- |
| type IV collagenase | C5138 | Sigma, USA |
| stem cell culture medium | HUXES-90011 | Cyagen, China |
| EGF | AF-100-15-500 | PeproTech, USA |
| A8301 | M5037 | AbMole, USA |
| Noggin | 120-10C-20 | PeproTech, USA |
| SB202190 | M2062 | AbMole, USA |
| prostaglandin E2 | M5929 | AbMole, USA |
| B27 | 12587010 | Gibco, USA |
| Matrigel | 356231 | BD, USA |
| TrypLE™ Express Enzyme | 12604021 | Thermo Scientific, USA |

**Table S3 Antibodies for Flow cytometry analysis**

| **Antibody** | **Catalog** | **Manufacturer** |
| --- | --- | --- |
| EpCAM (APC) | 324207 | Biolegend, USA |
| CEA CAM1 (PerCP/Cy5.5) | 342311 | Biolegend, USA |
| CD31 (PE) | 560983 | BD Biosciences, USA |
| CD45 (FITC) | 560976 | BD Biosciences, USA |
| CD133 (APC) | 372805 | Biolegend, USA |
| CD166 (PE) | 343903 | Biolegend, USA |

**Table S4 Antibodies for IHC analysis**

| **Antibody(Source)** | **Catalog** | **Dilution for IHC** | **Manufacturer** |
| --- | --- | --- | --- |
| Ki-67 (Rabbit) | ab92742 | 1:500 | Abcam, UK |
| EpCAM (Rabbit) | ab223582 | 1:500 | Abcam, UK |
| MUC2 (Rabbit) | ab133555 | 1:200 | Abcam, UK |
| α-SMA (Rabbit) | ab32575 | 1:500 | Abcam, UK |
| CD68 (Mouse) | ab201340 | 1:200 | Abcam, UK |

**Table S5 Antibodies for Immunofluorescence assays**

| **Antibody(Source)** | **Catalog** | **Dilution for IF** | **Manufacturer** |
| --- | --- | --- | --- |
| E-Cadherin (Mouse) | 14472 | 1:100 | CST, USA |
| β-catenin (Rabbit) | A19657 | 1:50 | Abclonal, China |
| Villin (Rabbit) | ab109516 | 1:100 | Abcam, UK |
| Ki-67 (Rabbit) | ab92742 | 1:100 | Abcam, UK |
| EpCAM (Rabbit) | ab223582 | 1:100 | Abcam, UK |

**Table S6 Primer sequences for qPCR**

| **Gene** | **Sequence** |
| --- | --- |
| CASP3 | F: ACACAGTATGGCGGCAGAG  R: AGACAGGCAACAGAGCACAT |
| CASP6 | F: GTTCTGAGCCTCGTGGTTATTC  R: AGCAATTCCTCTCCTCCTGTG |
| CASP7 | F: AAGCATTGTCTGTGGTTGTGTA  R: CAGCCAGGAGCAAGGAGAA |
| CASP9 | F: TTCTCGCTGCTCCGTTCTT  R: ACTGACTGATGGCTCCTTCC |
| BCL2 | F: TGTGAAGTGCTGCTCTATGGT  R: CCGTGGTGAACTGGATGCTA |
| BAX | F: CTTCAGGACACAGGACTCTCAG  R: CGCATCCACCGCACACTAA |
| PARP1 | F: CTTCCTGCCTCCTGTTAGTCA  R: GCTGCTGTCACCTTCATAGTAG |
| BIRC5 | F: GAGTGGATGTGGAGGTGTGA  R: TTCTGGAACTGGATGGTGTCA |
| TNF | F: TCAGCAAGGACAGCAGAGG  R: GCCACGATCAGGAAGGAGAA |
| GAPDH | F: ACCACAGTCCATGCCATCAC  R: TCCACCACCCTGTTGCTGTA |
| BCL2 promoter | F: CACACTCACACGGCCAGAA  R: AAGAAGAAAGAAAGAGCCCTCCTCT |

**Table S7 Antibodies for western blotting**

| **Antibody(Source)** | **Catalog** | **Dilution for wb** | **Manufacturer** |
| --- | --- | --- | --- |
| KLF5 (Rabbit) | 51586 | 1:1000 | CST, USA |
| Cleaved Caspase-3 (Rabbit) | 9661 | 1:1000 | CST, USA |
| Caspase-3 (Rabbit) | 9662 | 1:1000 | CST, USA |
| Bcl-2 (Rabbit) | ab32124 | 1:1000 | Abcam, UK |
| Bax (Rabbit) | ab32503 | 1:1000 | Abcam, UK |
| GAPDH (Mouse) | 60004-1-Ig | 1:1000 | Proteintech, USA |

**Table S8 siRNA Sequences**

| siRNA | **Stem Sequences** |
| --- | --- |
| BCL2-siRNA#1  **BCL2-siRNA#2**  BCL2-siRNA#3 | GTGATGAAGTACATCCATTAT  **TGGATGACTGAGTACCTGAAC**  CCGCATTTAATTCATGGTATT |
| **Bax-siRNA#1**  Bax-siRNA#2  Bax-siRNA#3 | **GACGAACTGGACAGTAACATG**  TTCTACTTTGCCAGCAAACTG  CGAGTGGCAGCTGACATGTTT |

***Bold fonts represent the most effective sequence**

**Table S9 shRNA Sequences**

| shRNA | **Stem Sequences** |
| --- | --- |
| KLF5-LV3-shRNA #1  **KLF5-LV3-shRNA #2**  KLF5-LV3-shRNA #3 | GCTGTAATGTATATGGCTTTA  **ACAAATAGCCATTGAACAAAT**  AGGTAATTCCTTAGAGATACA |

***Bold fonts represent the most effective sequence**

**Table S10 Patient information of oxaliplatin sensitivity assay**

| Sample ID | Patient age | Patient sex | Tumor location | TNM stage | TRG |
| --- | --- | --- | --- | --- | --- |
| P1 | 63 | M | R | ypT4aN1 | 3 |
| P2 | 65 | M | R | ypT3N1 | 2 |
| P3 | 71 | M | R | ypT2N0 | 1 |
| P4 | 57 | F | R | ypT3N0 | 2 |
| P5 | 35 | M | R | ypT2N0 | 1 |
| P6 | 59 | M | R | pCR | 0 |
| P7 | 75 | M | R | ypT3N0 | 1 |
| P8 | 63 | F | R | pCR | 0 |
| P9 | 72 | M | R | ypT4bN1 | 3 |
| P10 | 54 | M | R | ypT4bN2 | 3 |
| P11 | 71 | F | R | ypT3N0 | 2 |
| P12 | 48 | M | R | pCR | 0 |

Tumor location: A, ascending colon; T, transverse colon; D, descending colon; S, sigmoid colon, R, rectum. TRG, Tumor Regression Grade.
