# Supplementary material for: KLF5 inhibition overcomes oxaliplatin resistance in patient-derived colorectal cancer organoids by restoring apoptotic response
Source: Cell Death Dis. 2022 Apr 5;13(4):303. doi: 10.1038/s41419-022-04773-1 (PMC8980070; doi:10.1038/s41419-022-04773-1)

Figure 4B KLF5

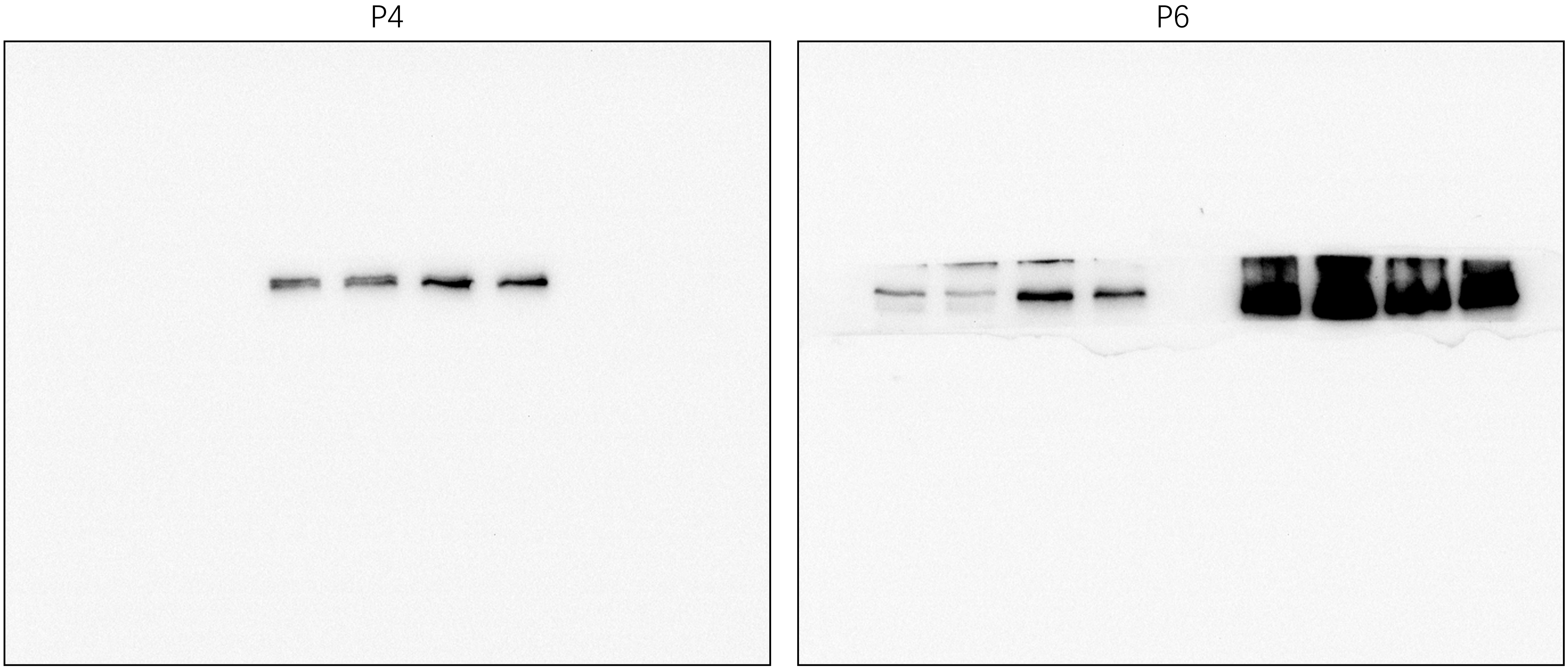

Figure 4B Bcl-2

P4

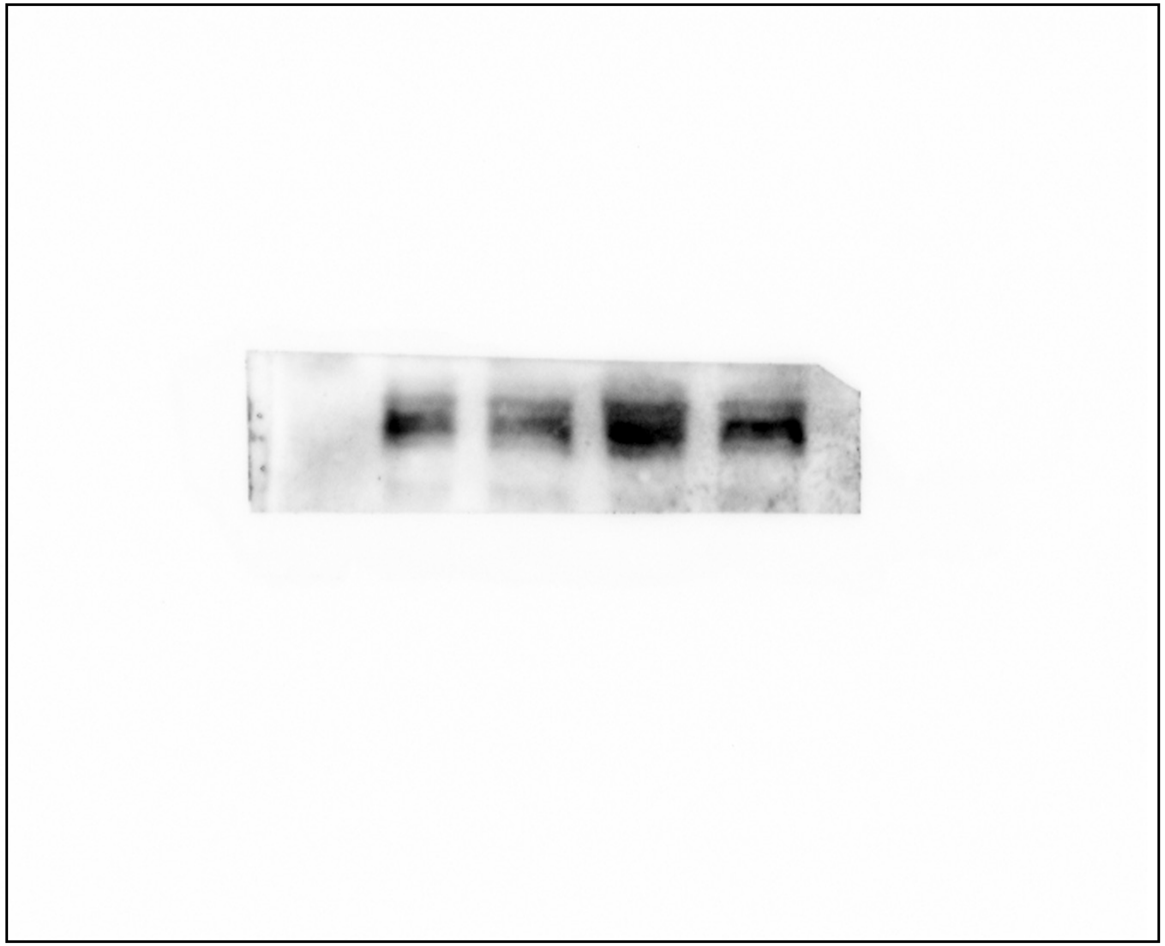

P6

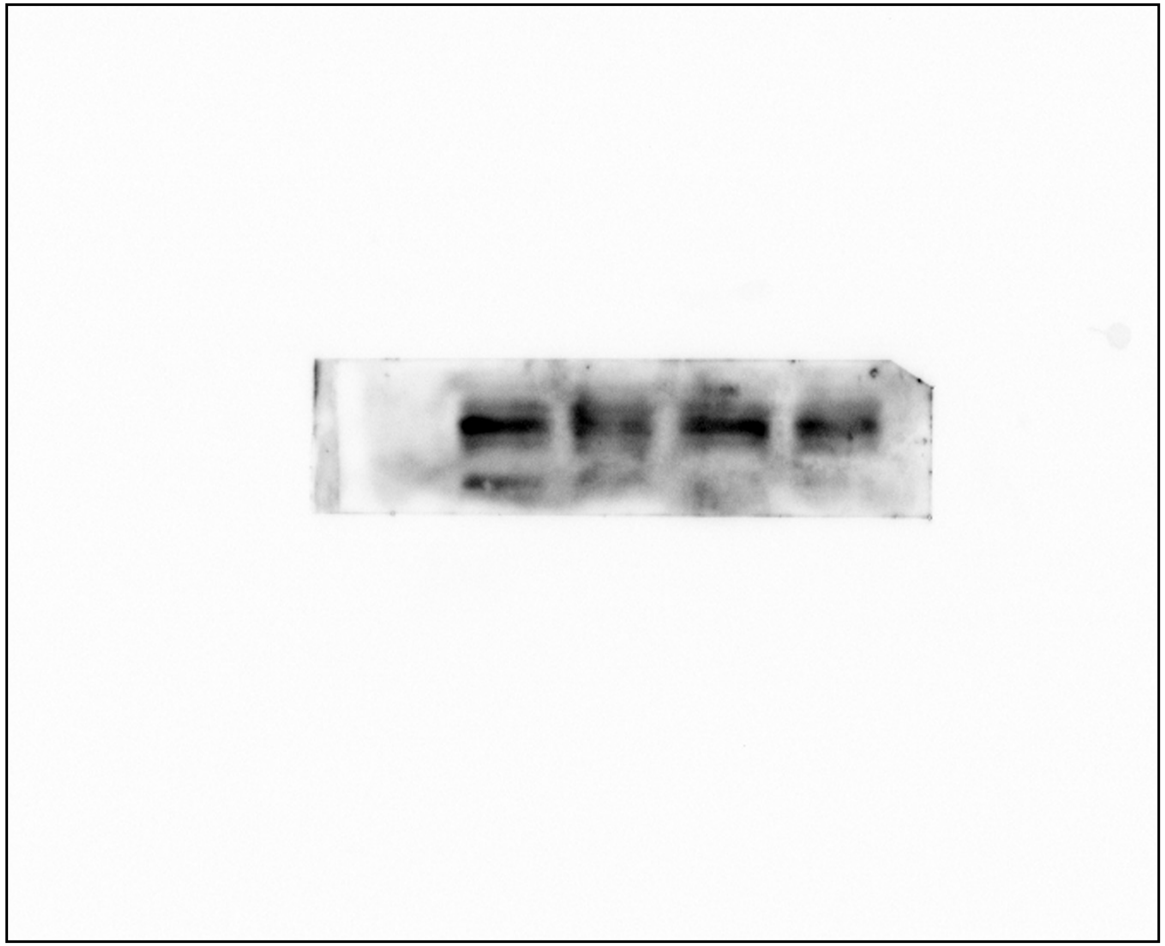

Figure 4B Bax

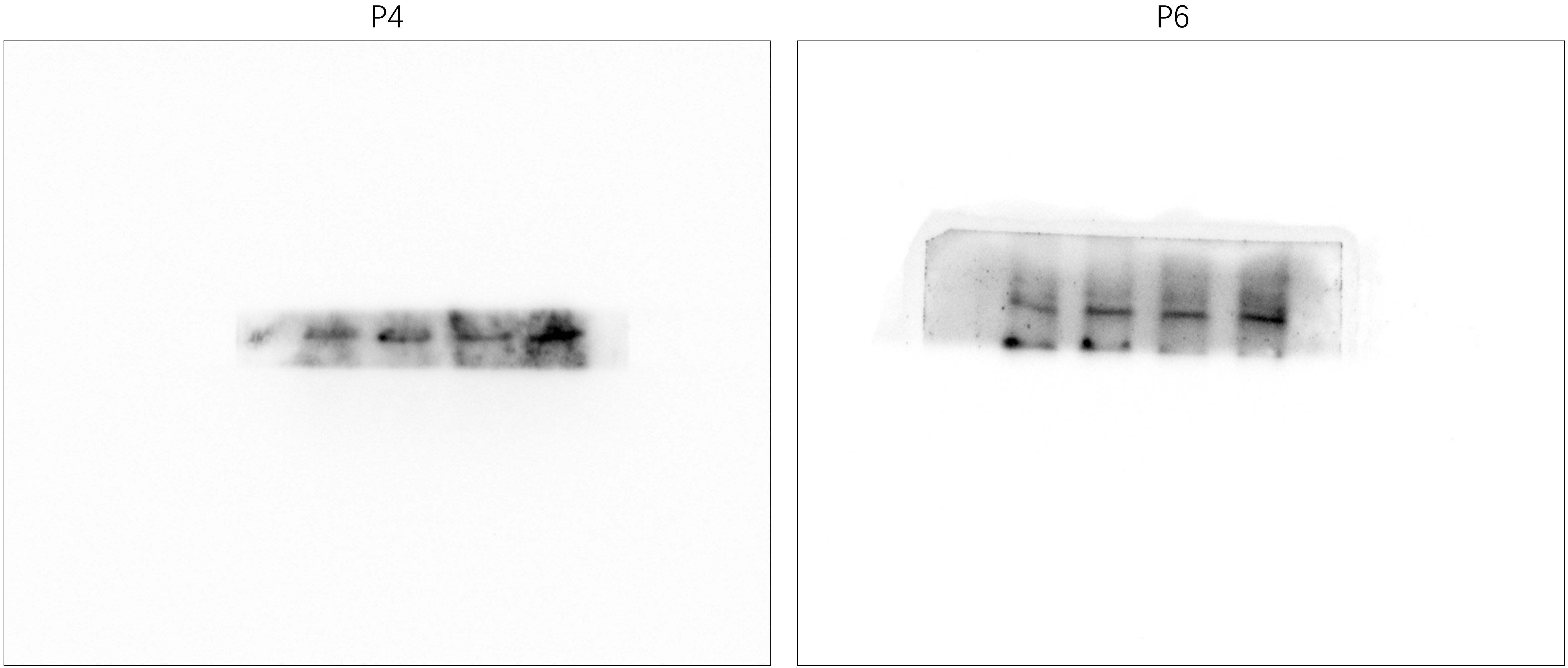

Figure 4B Cleaved Caspase-3

P4 P6

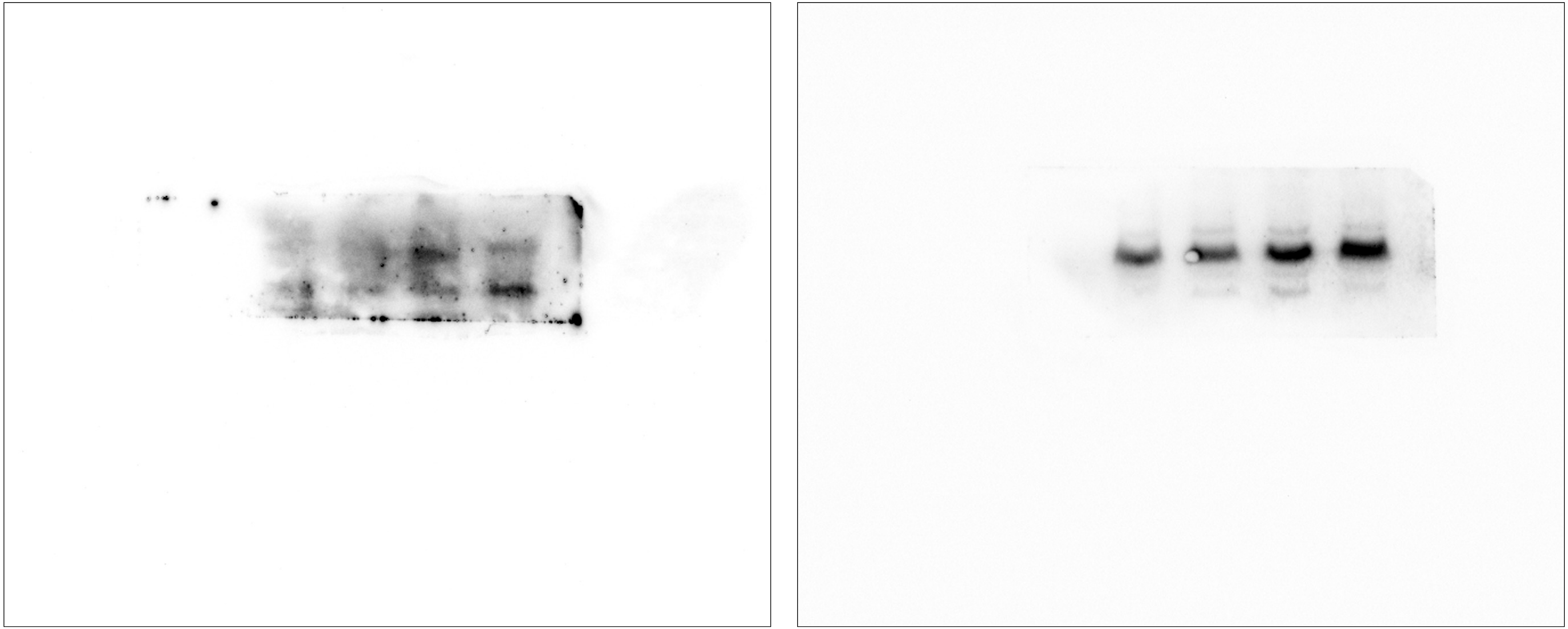

Figure 4B Caspase-3

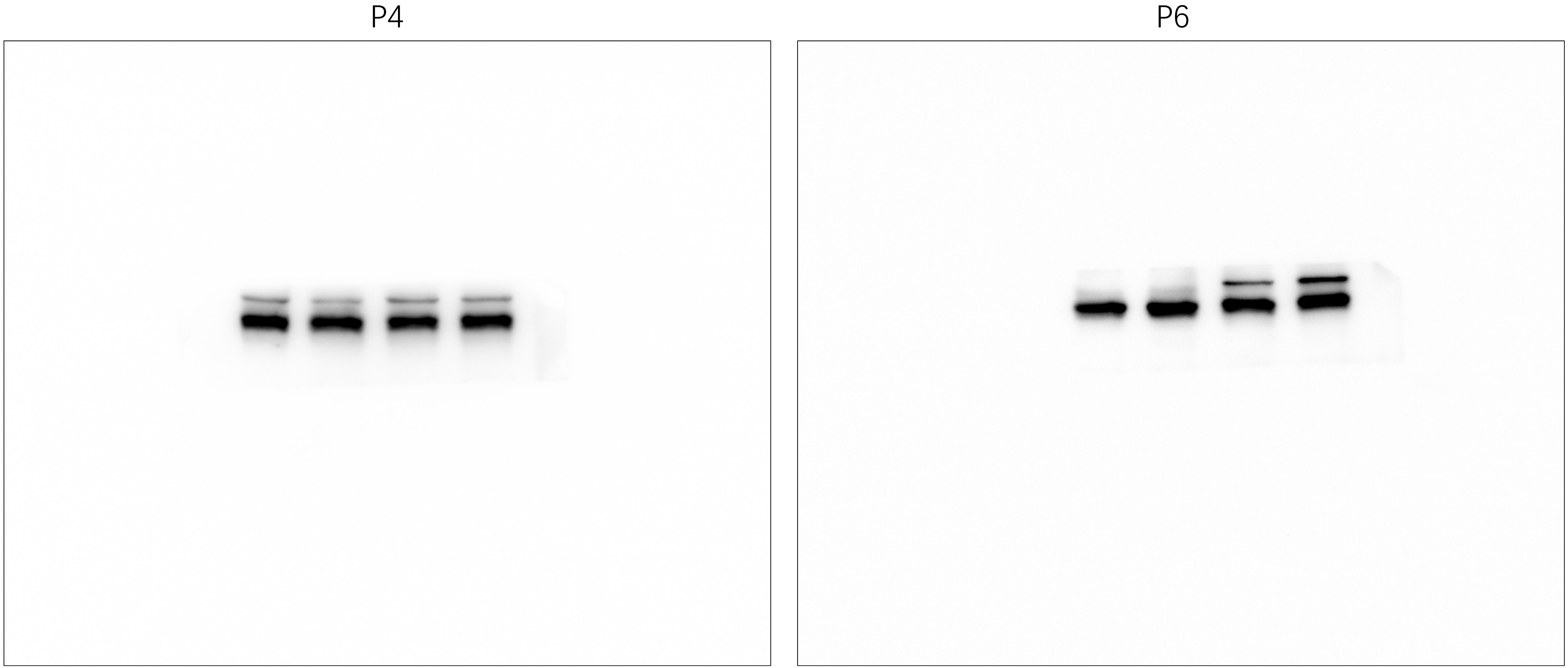

Figure 4B GAPDH

P4

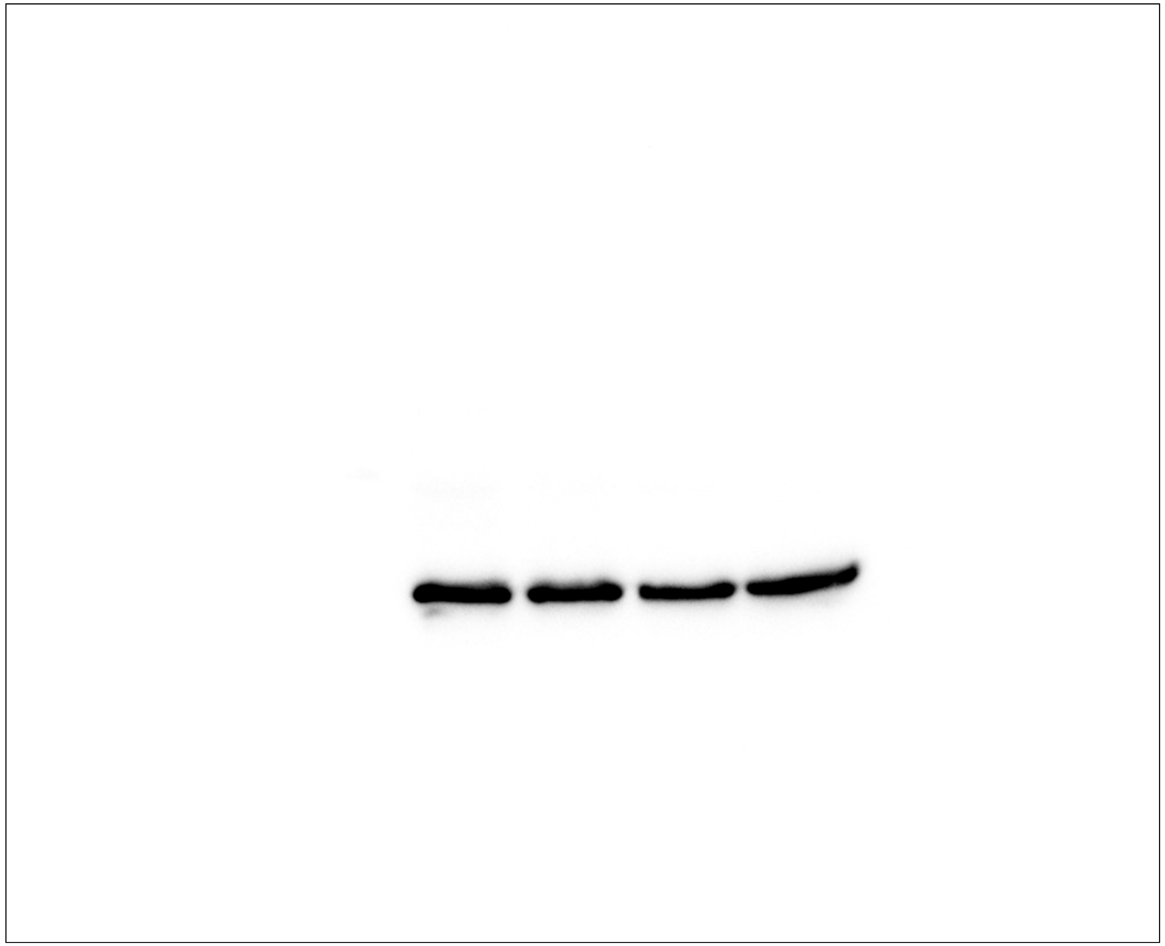

P6

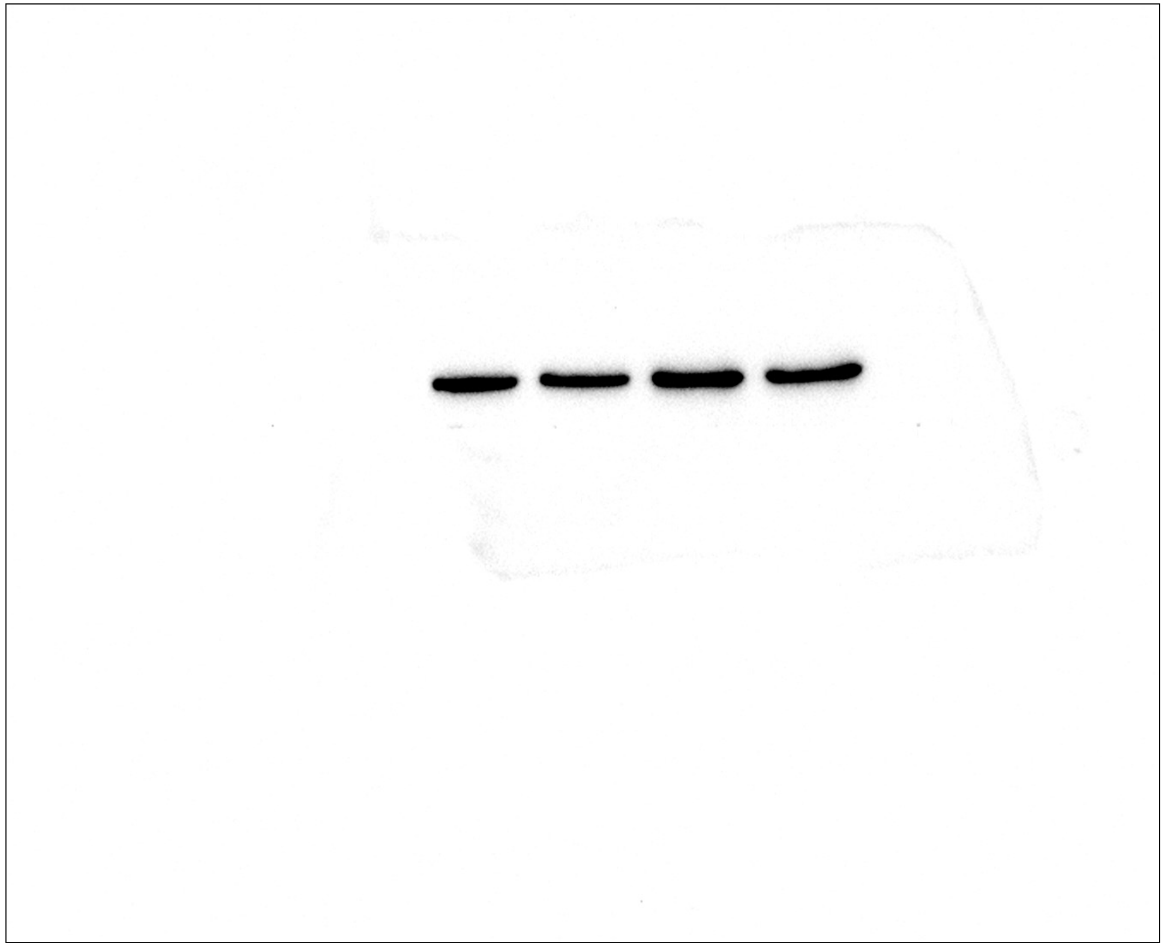

Figure 4F

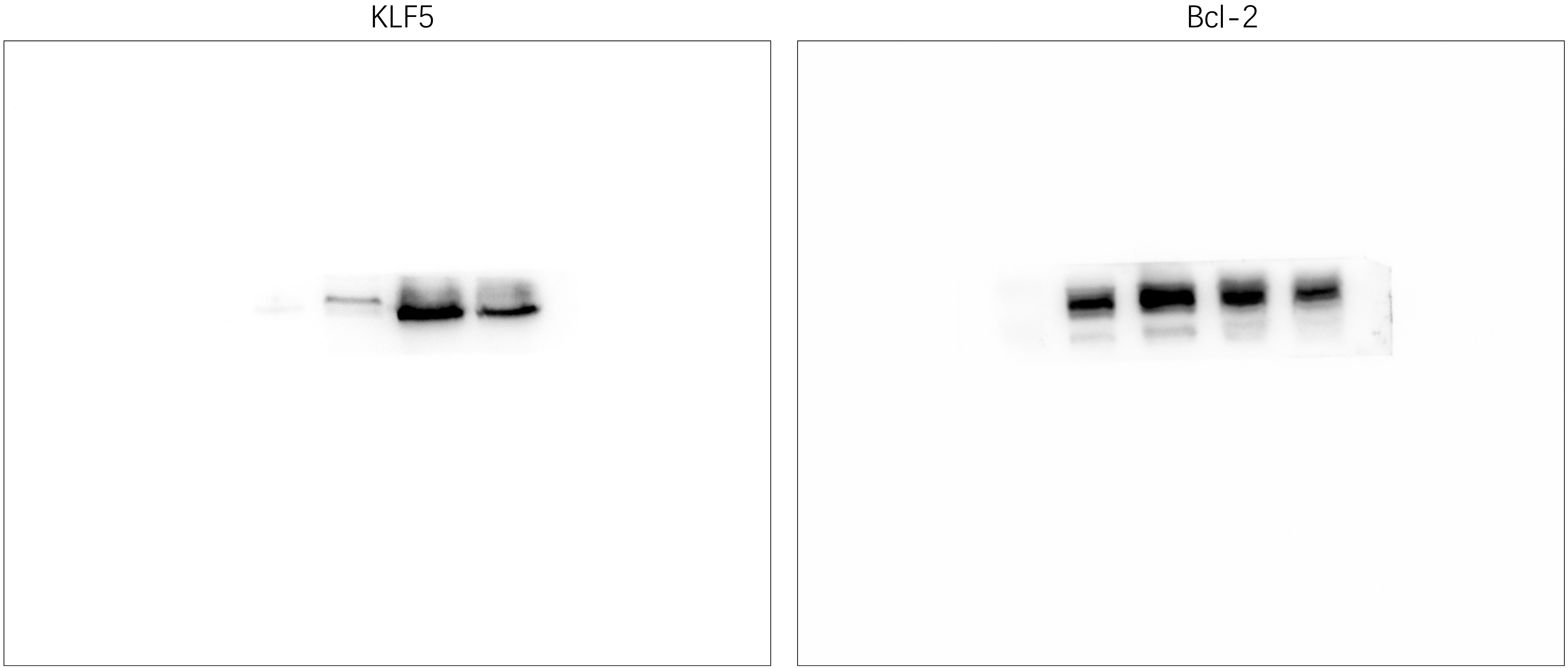

Figure 4F

Bax

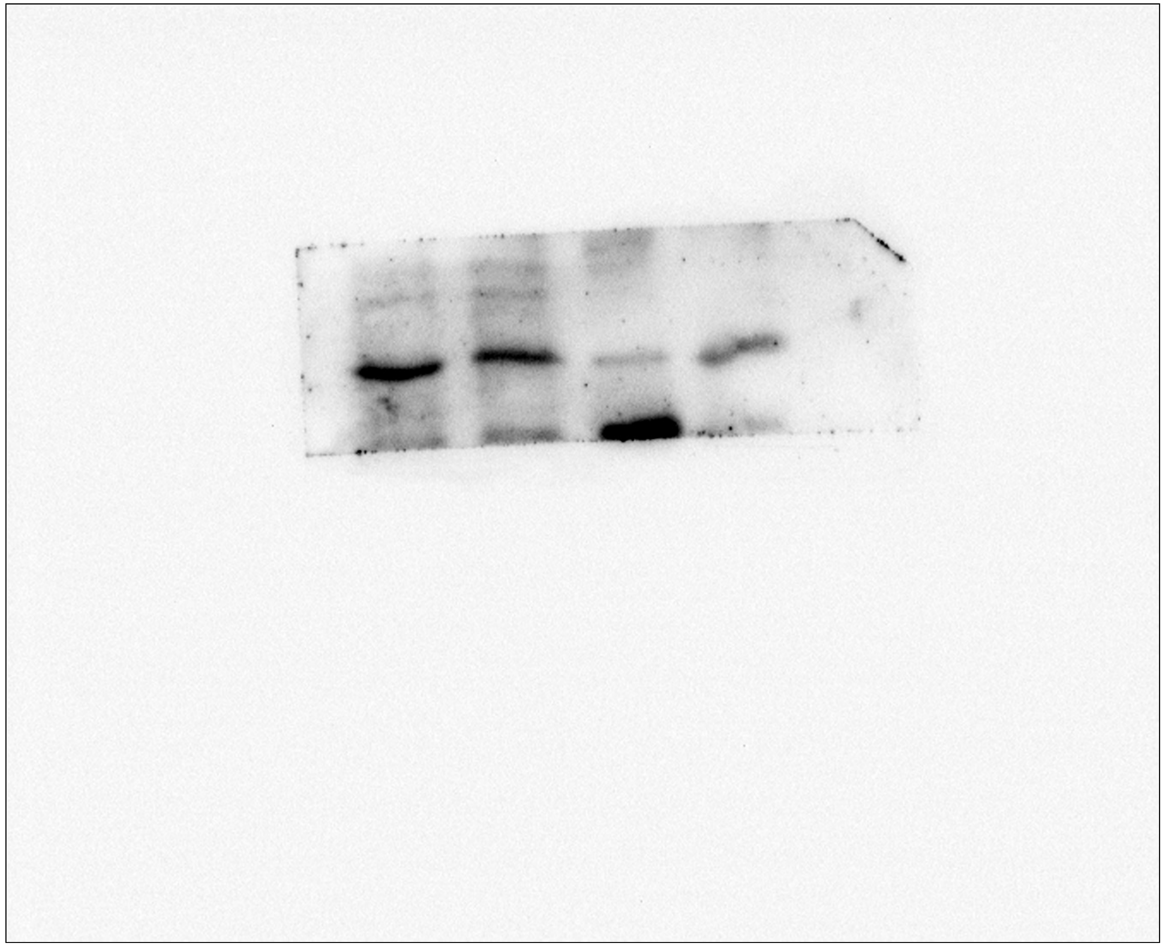

Cleaved Caspase-3

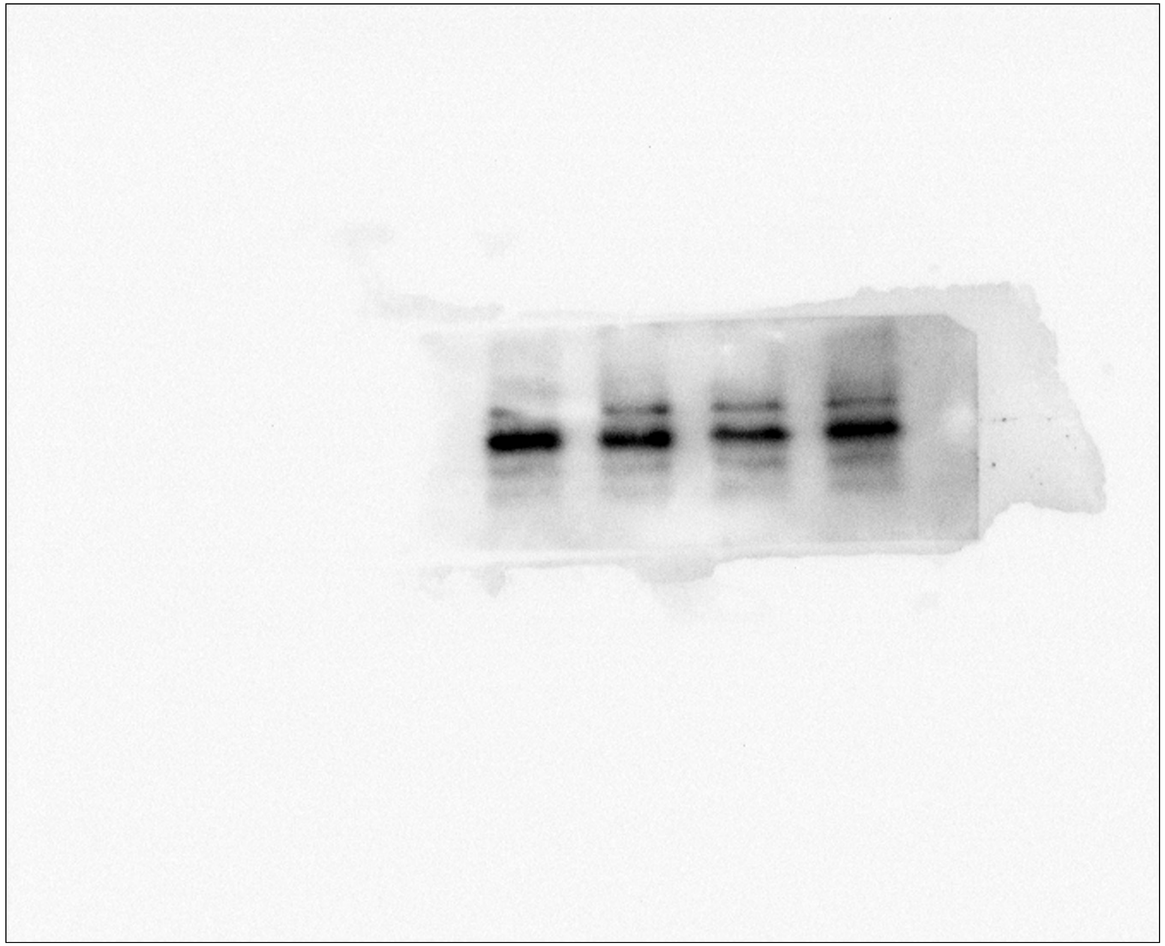

Figure 4F

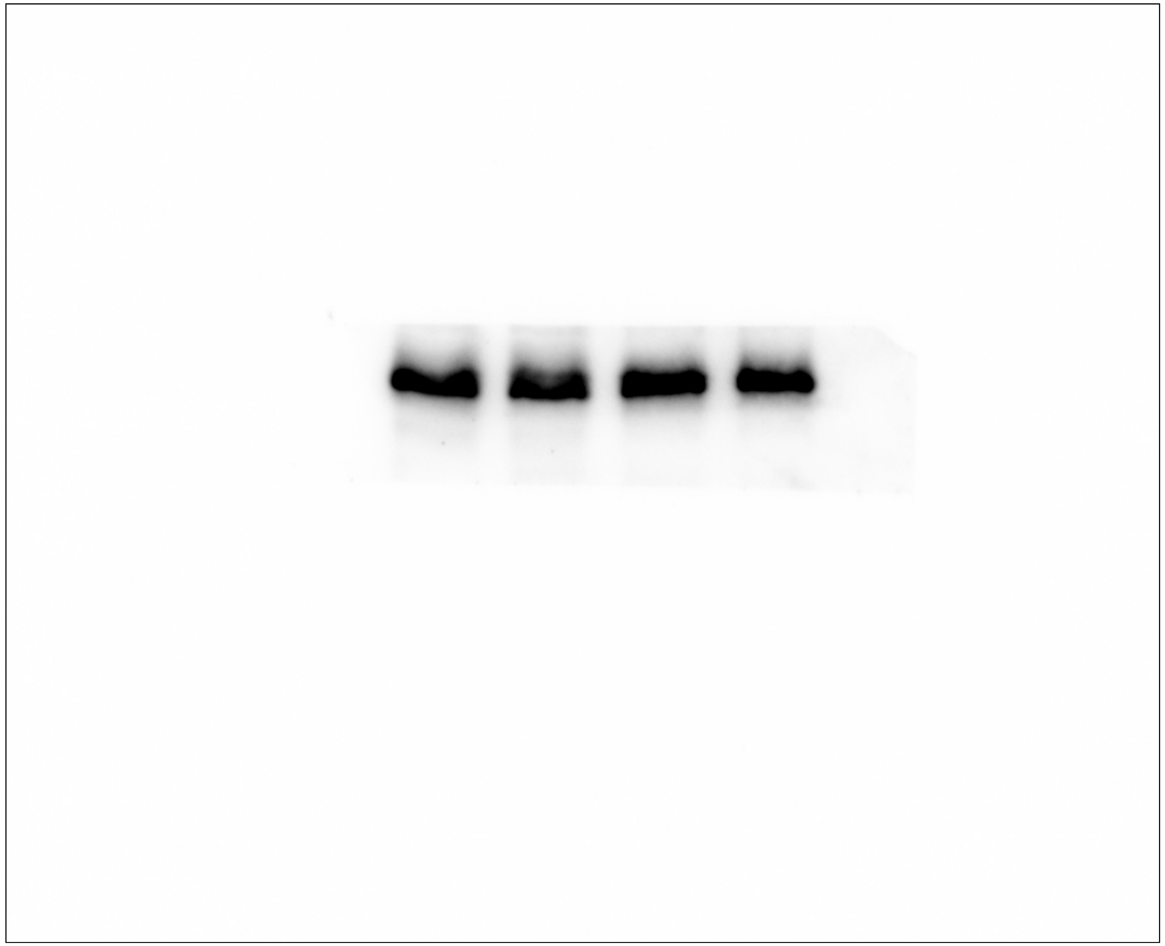

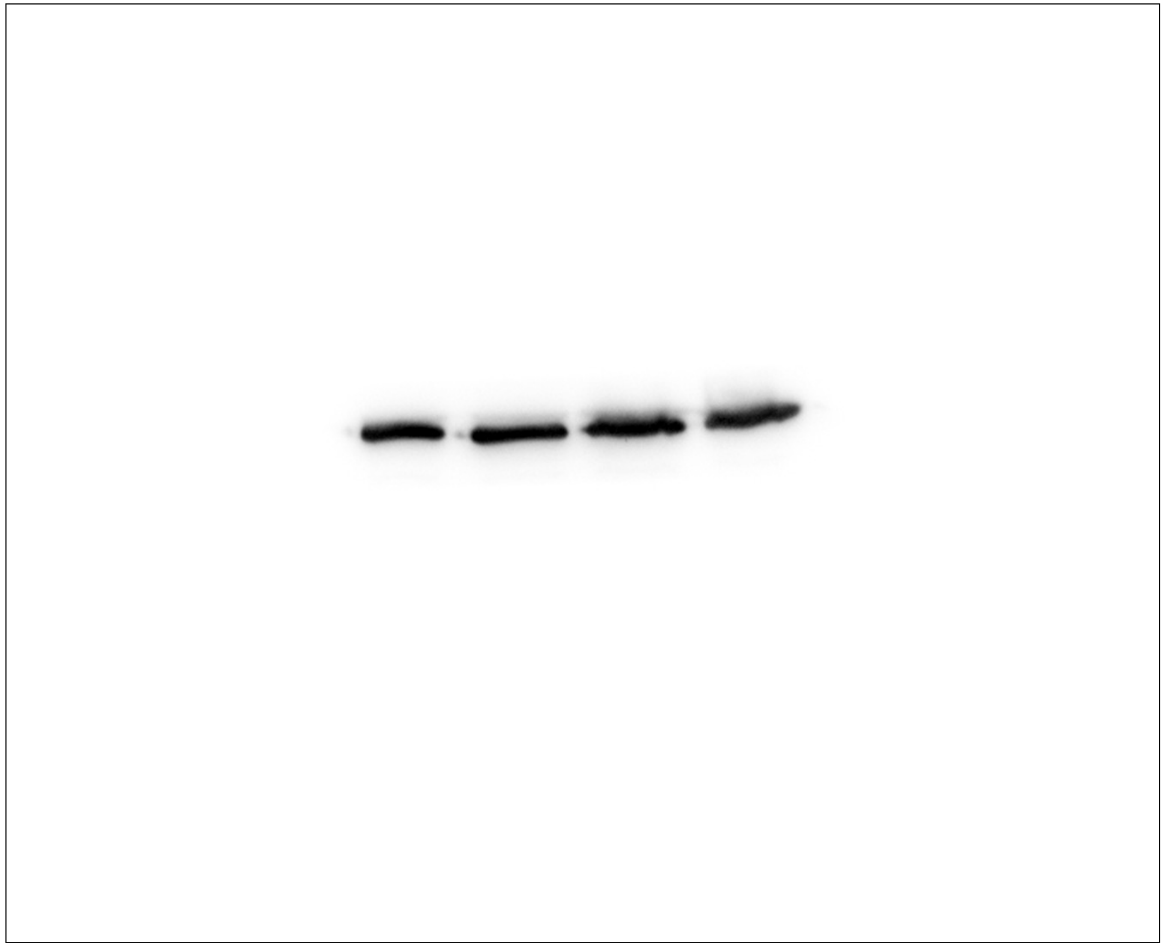

Figure 5A

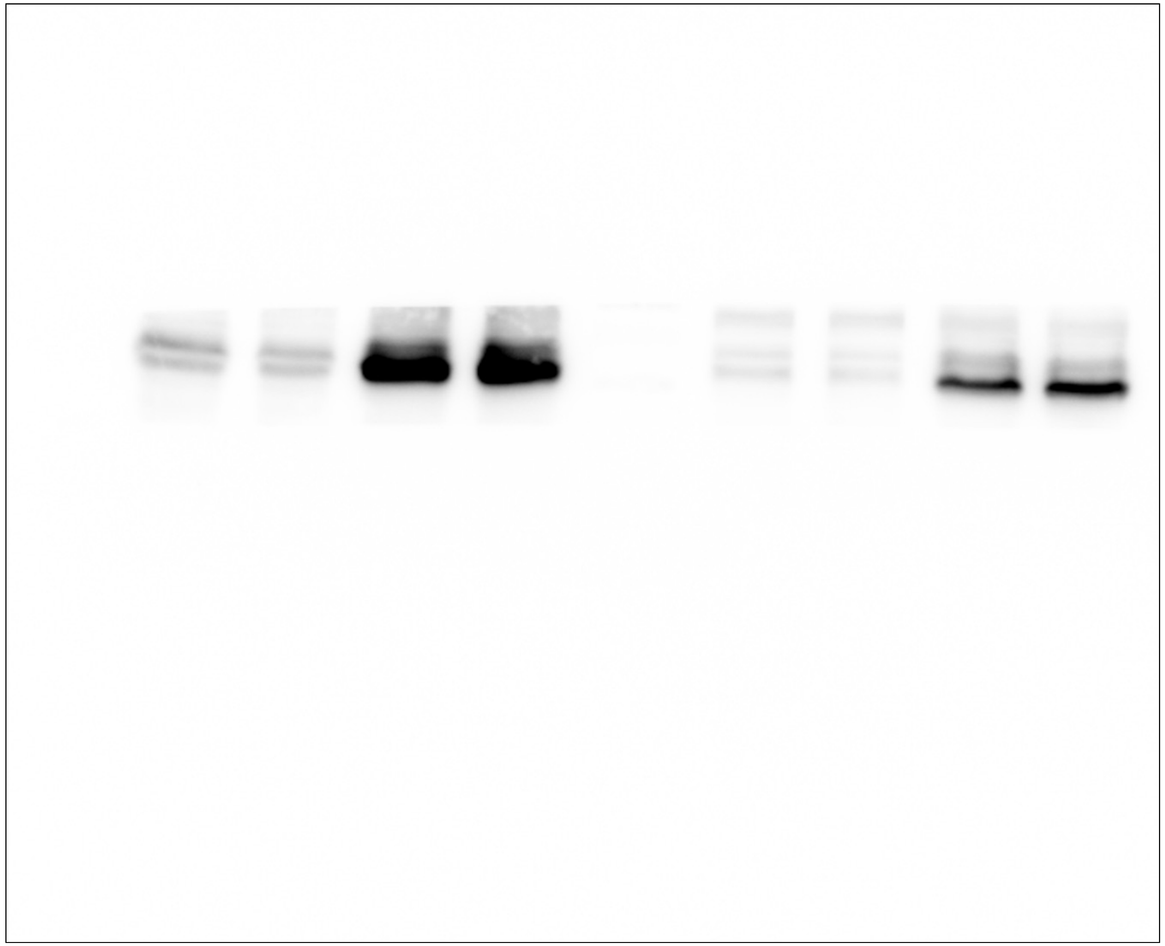

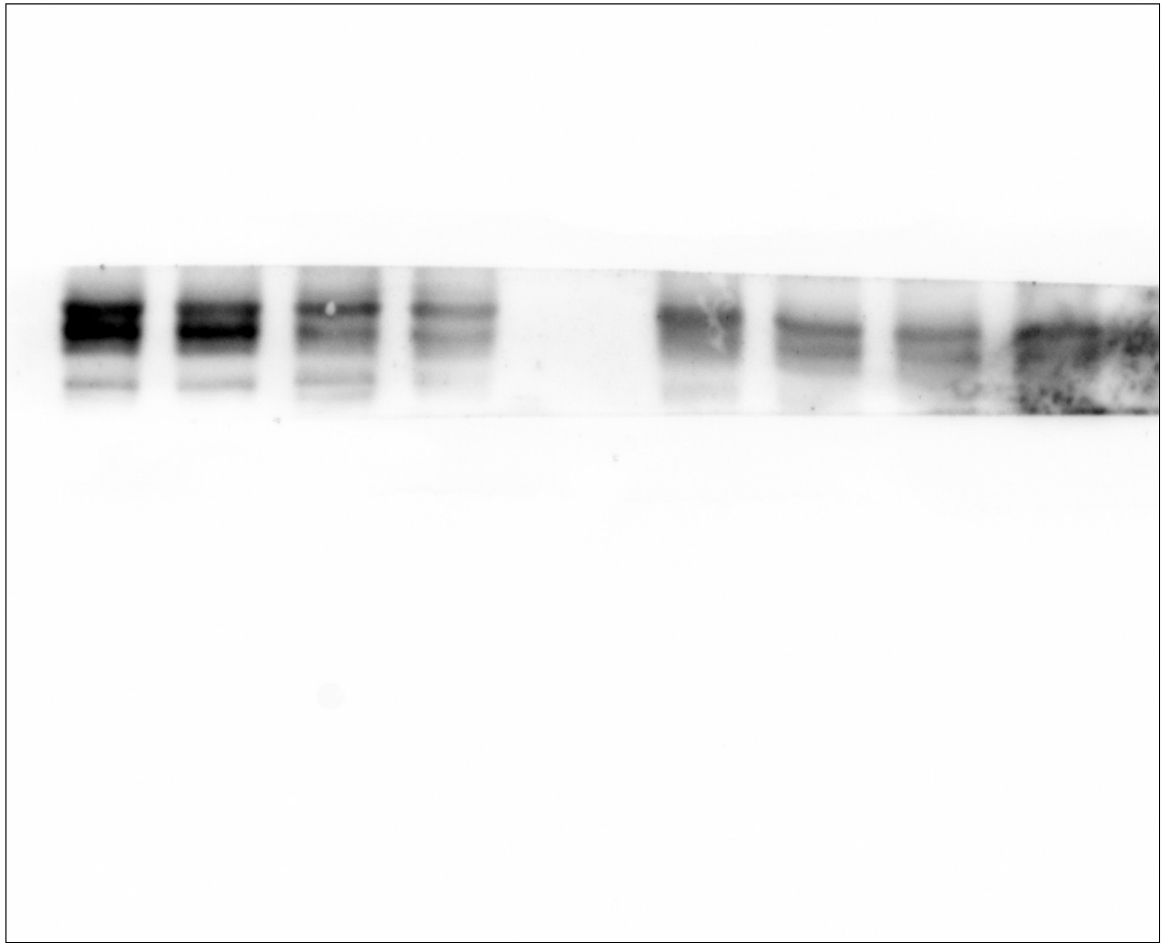

Figure 5A

Bax

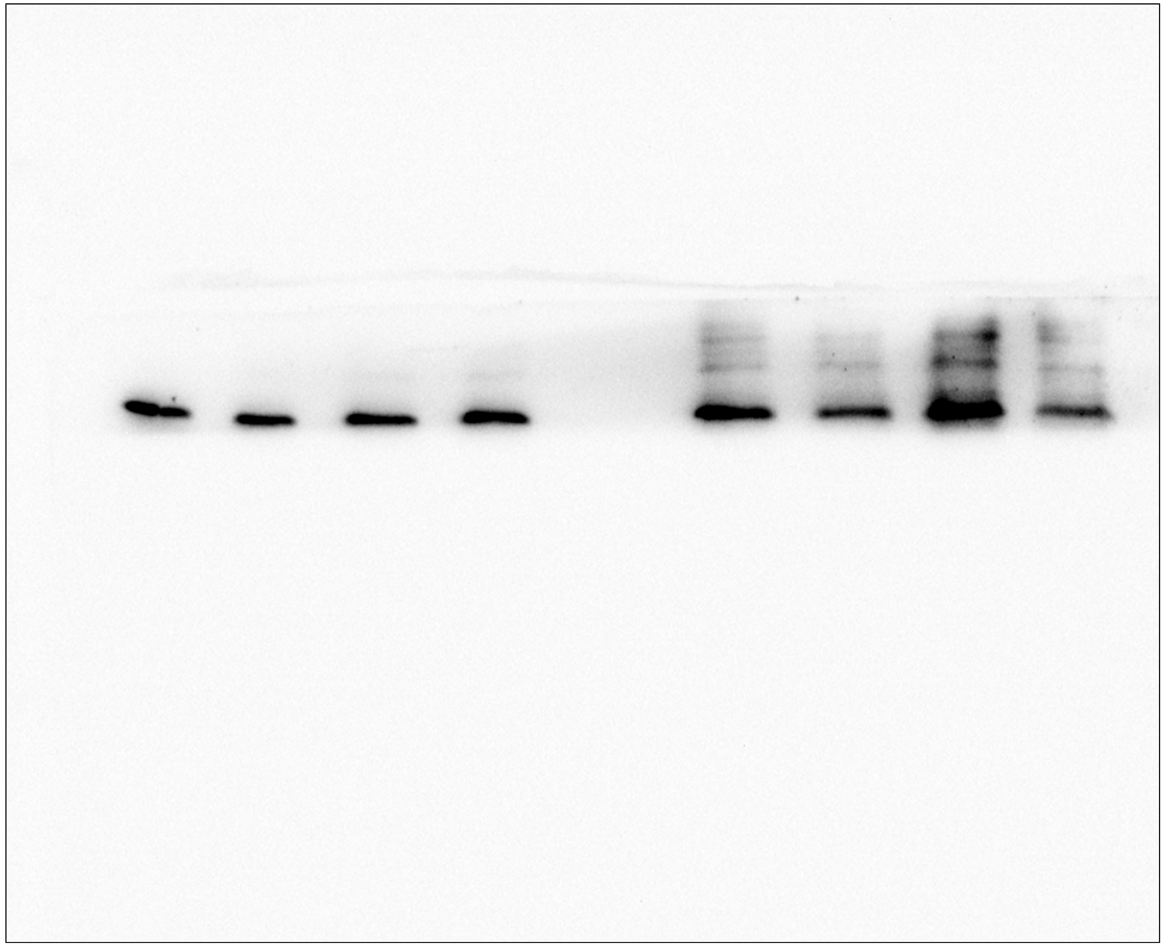

Cleaved Caspase-3

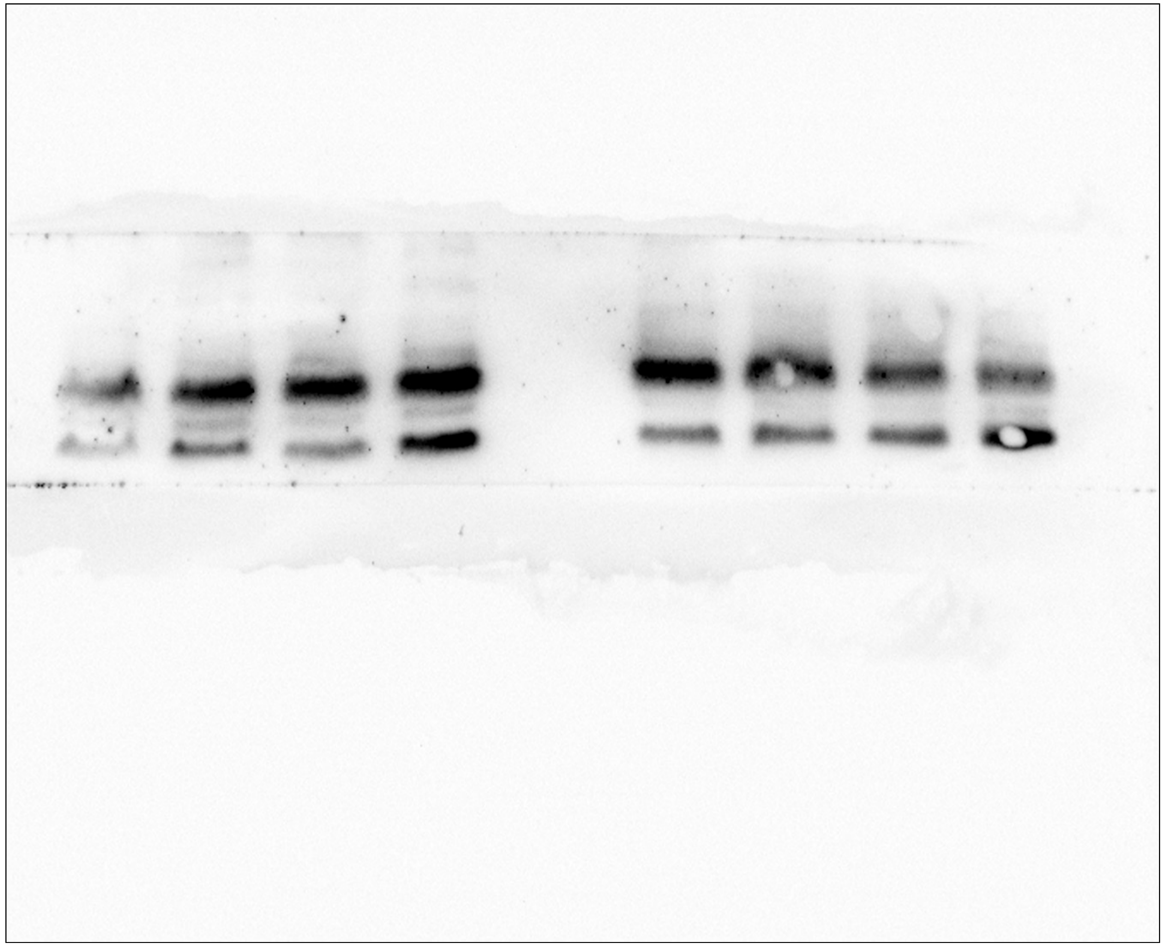

Figure 5A

GAPDH

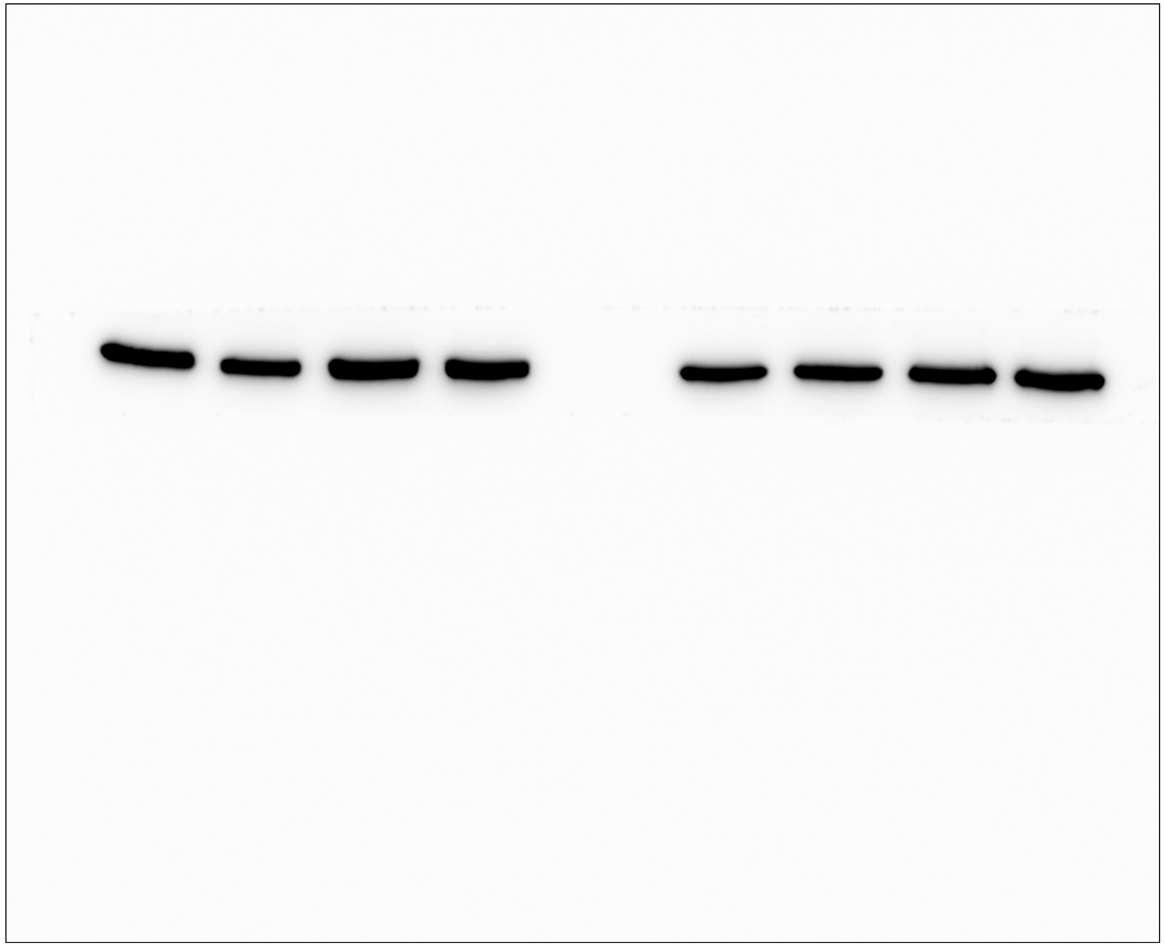

Figure 5C KLF5

RKO/Vector RKO/KLF5 SW620/sh-NC SW620/sh-KLF5

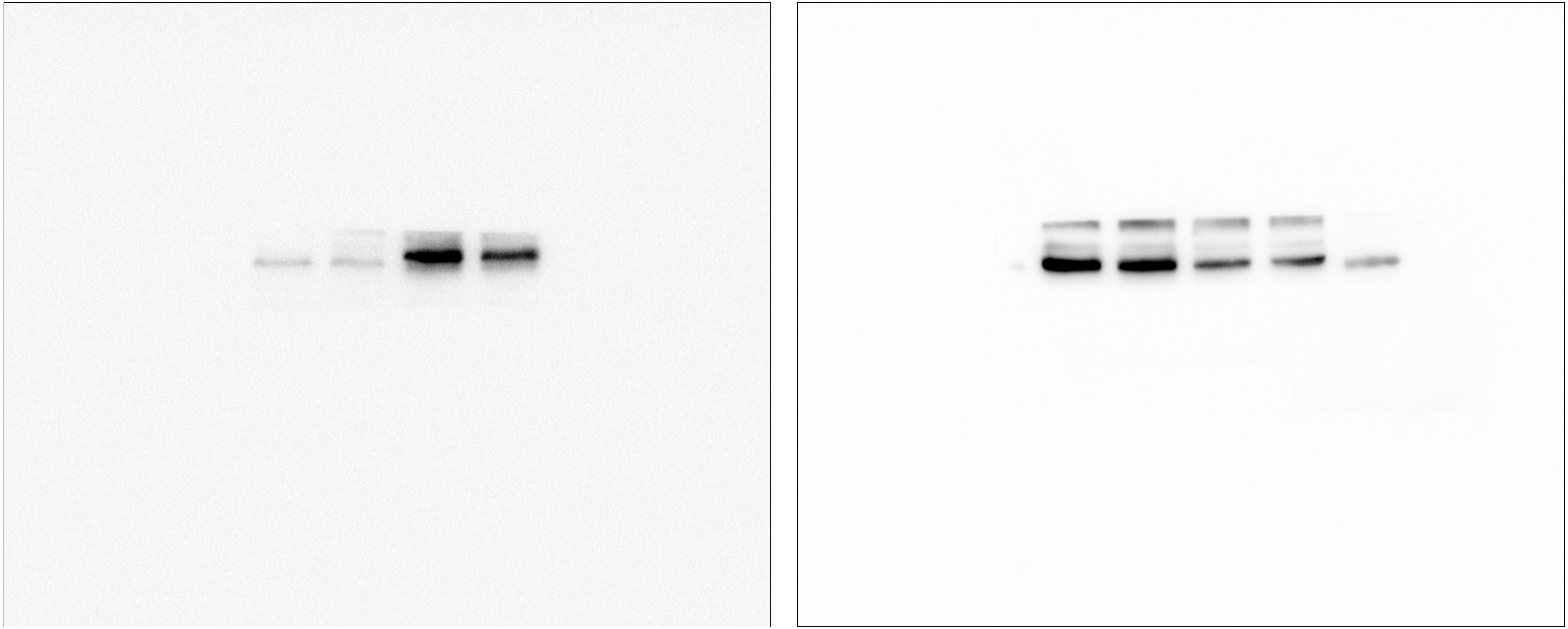

Figure 5C Bcl-2

RKO/Vector RKO/KLF5   SW620/sh-NC   SW620/sh-KLF5

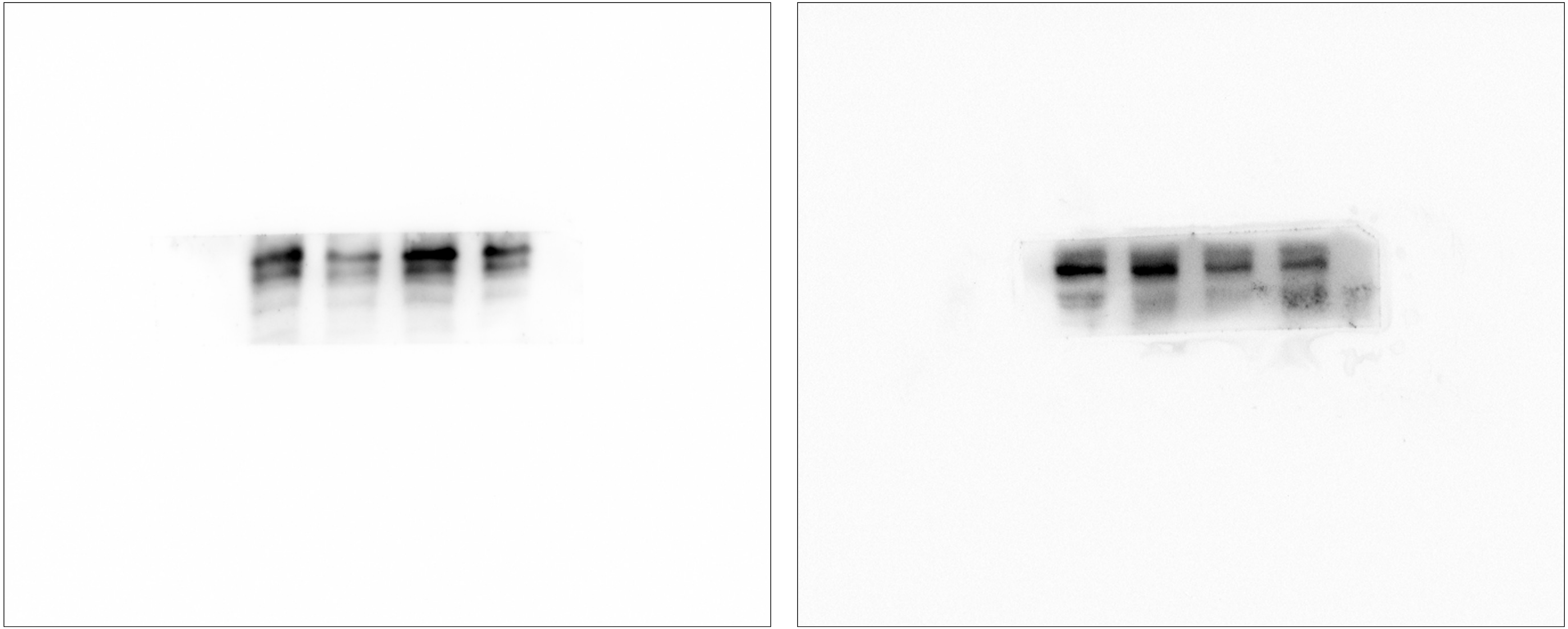

Figure 5C Bax

RKO/Vector RKO/KLF5

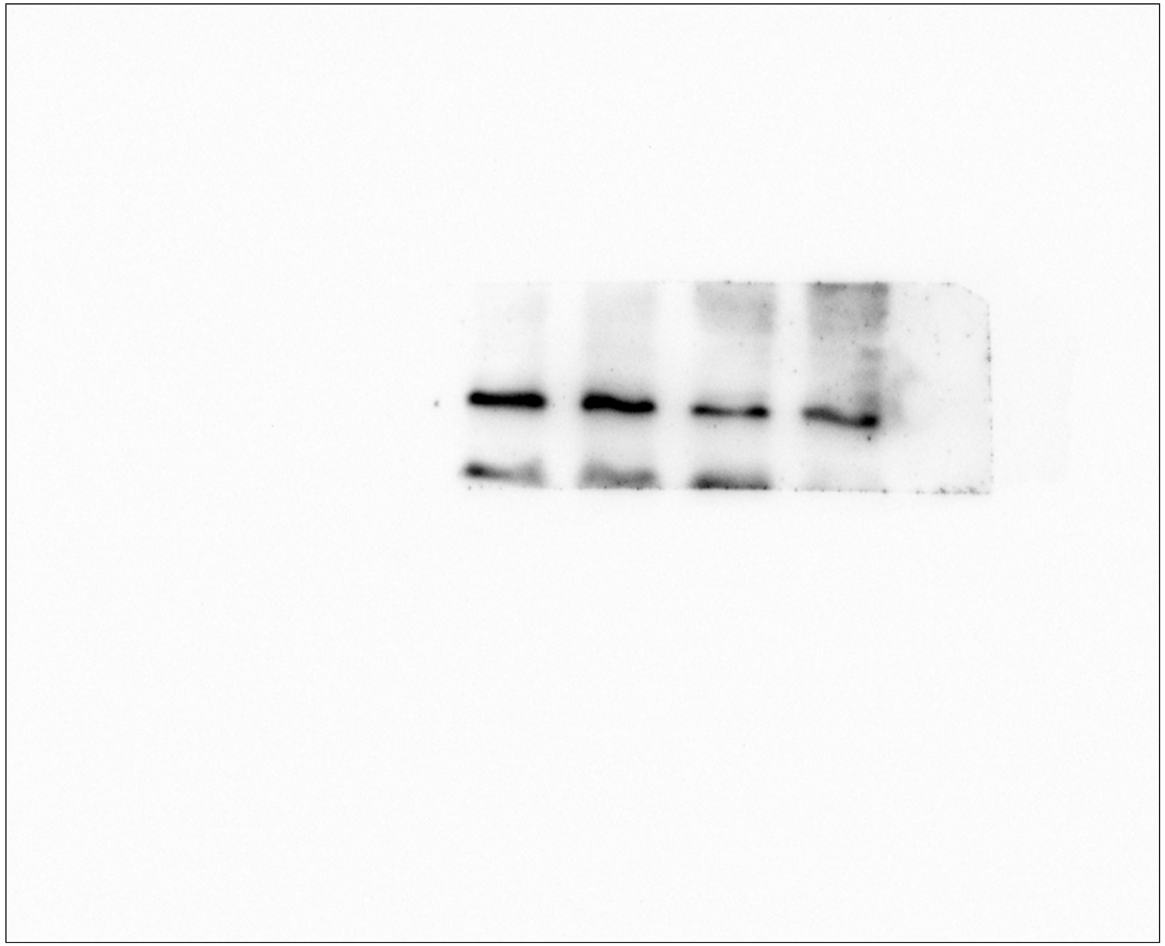

SW620/sh-NC   SW620/sh-KLF5

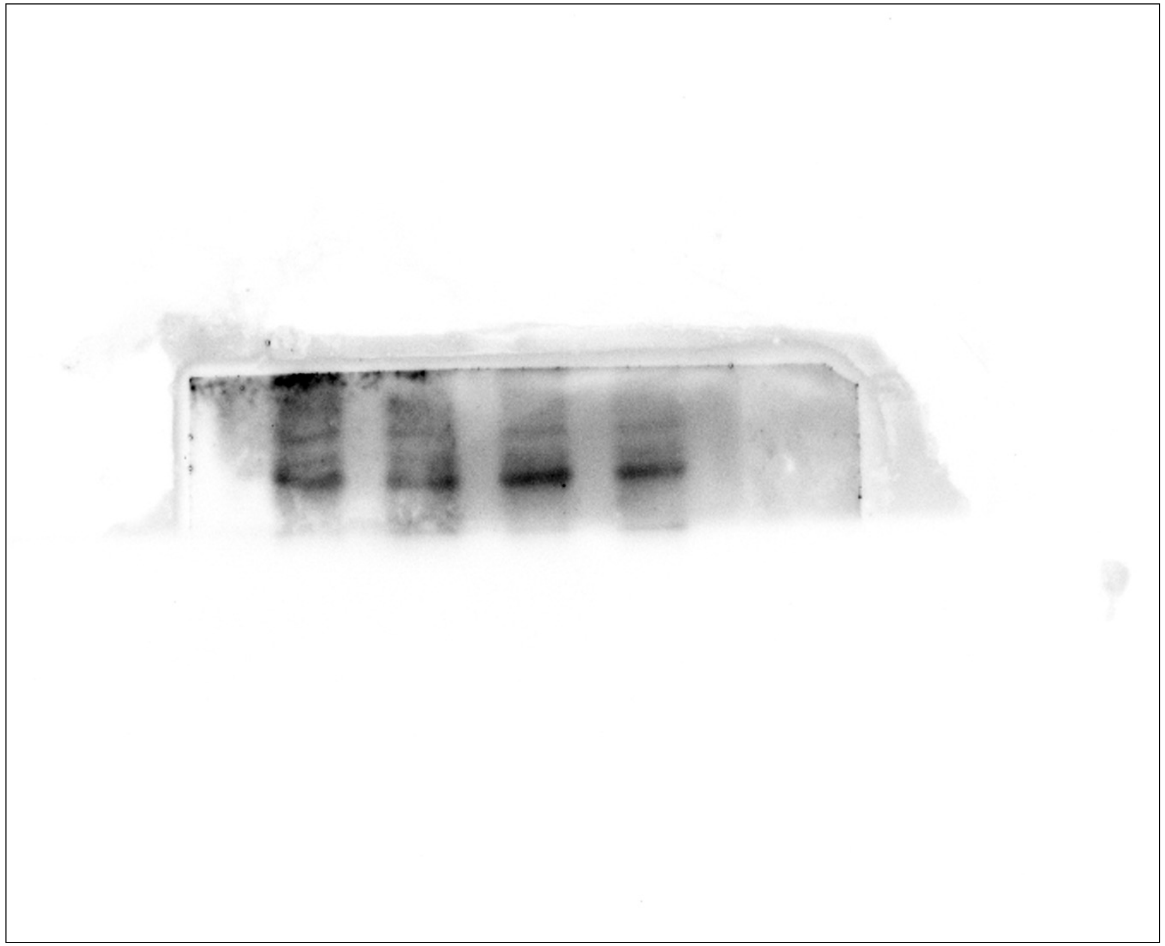

Figure 5C Cleaved Caspase-3

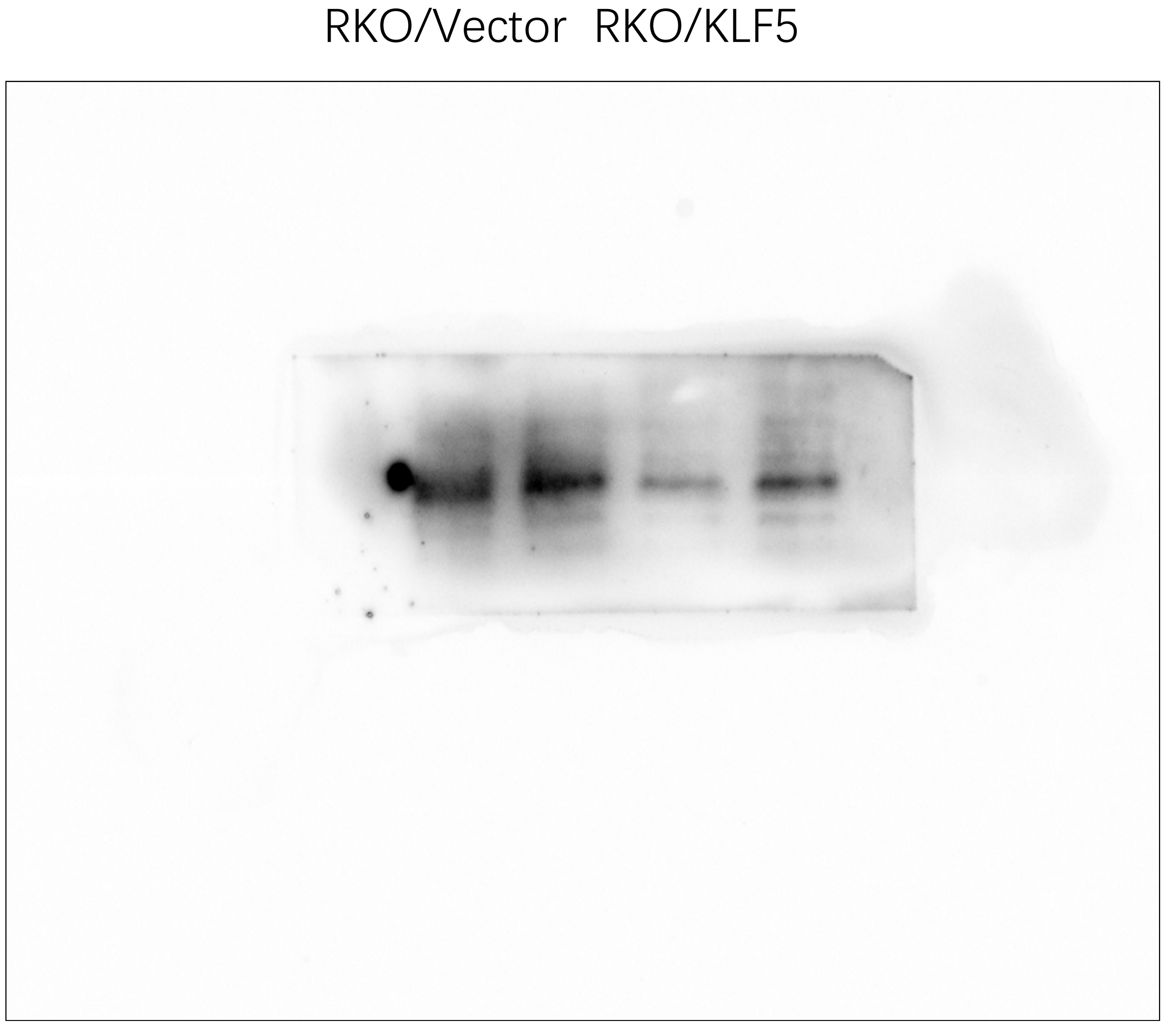

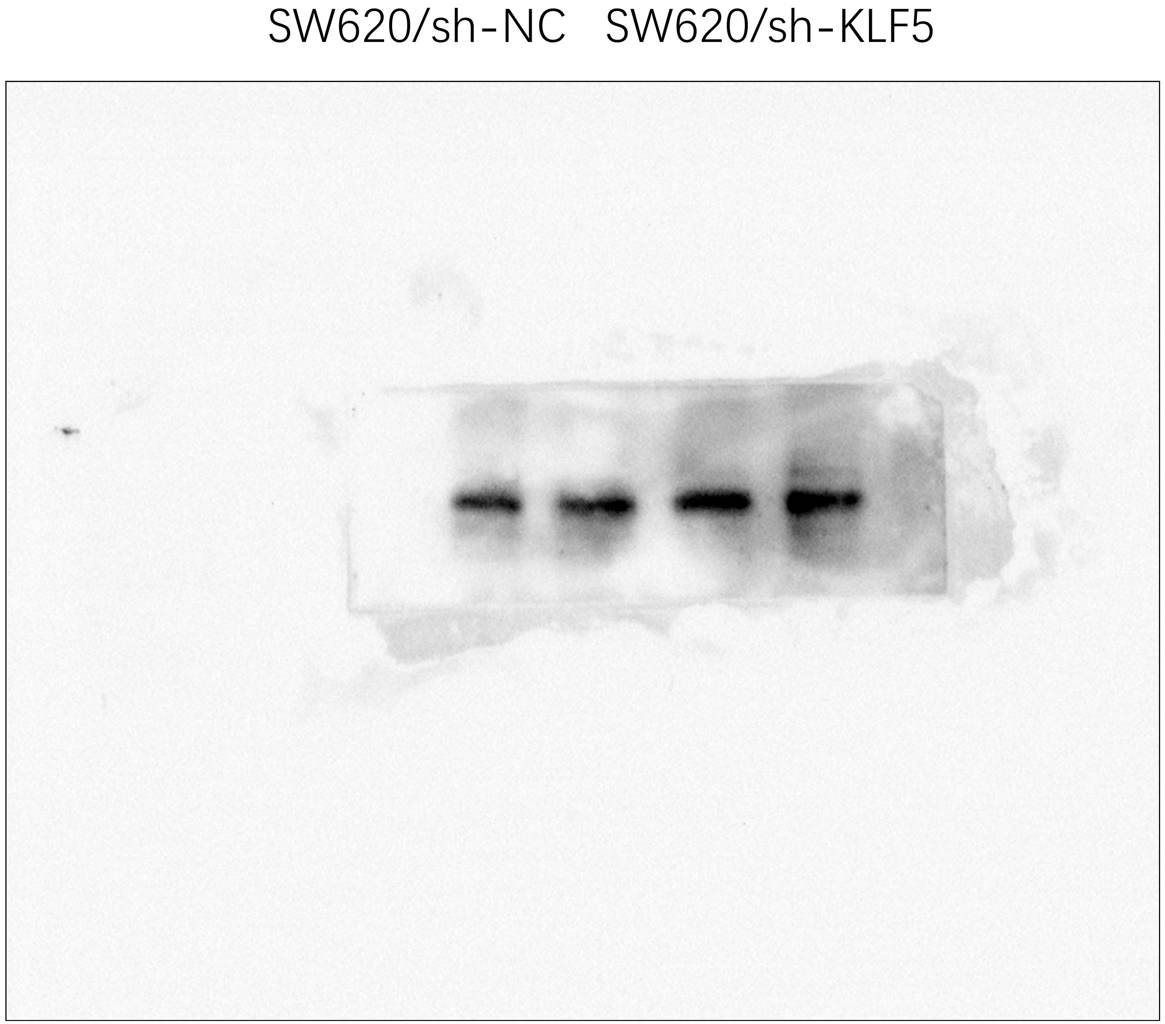

Figure 5C GAPDH

RKO/Vector RKO/KLF5

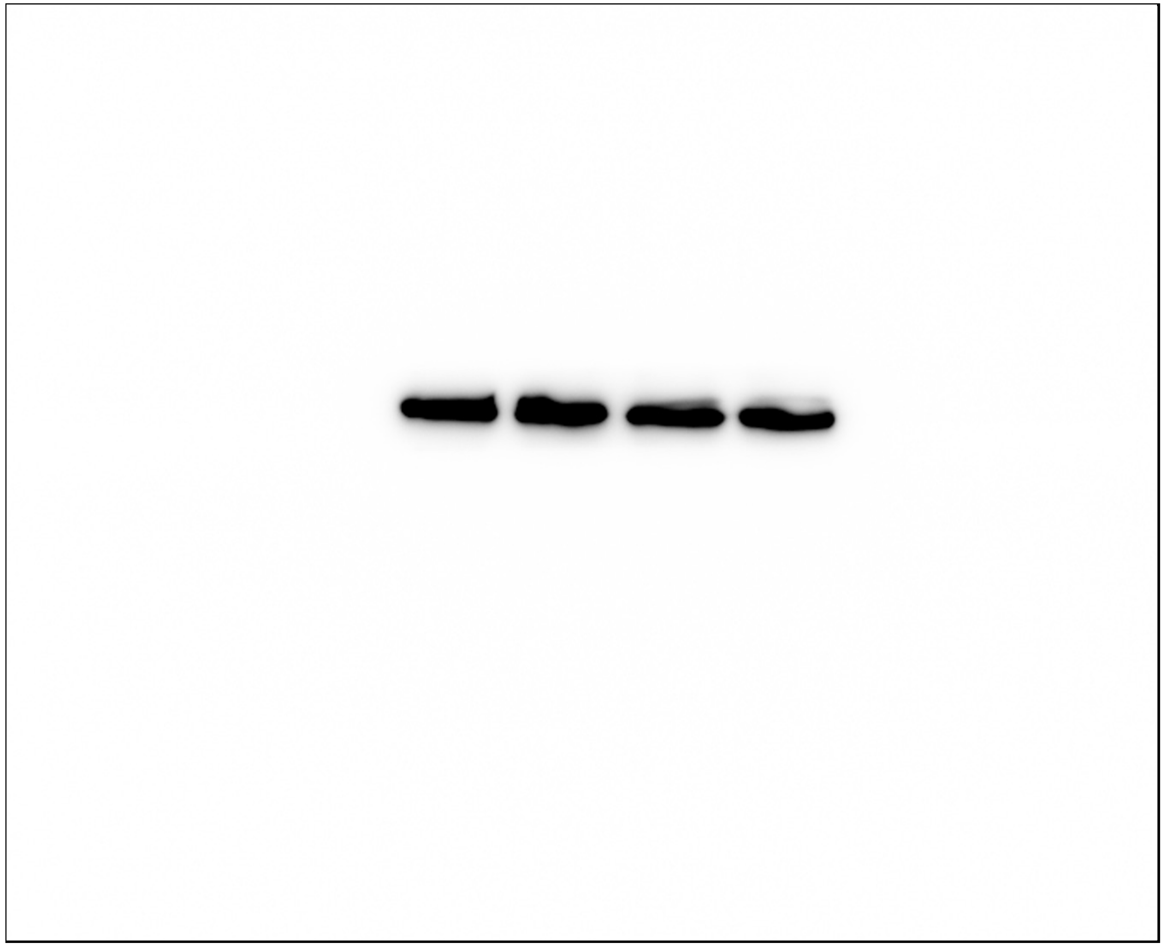

SW620/sh-NC SW620/sh-KLF5

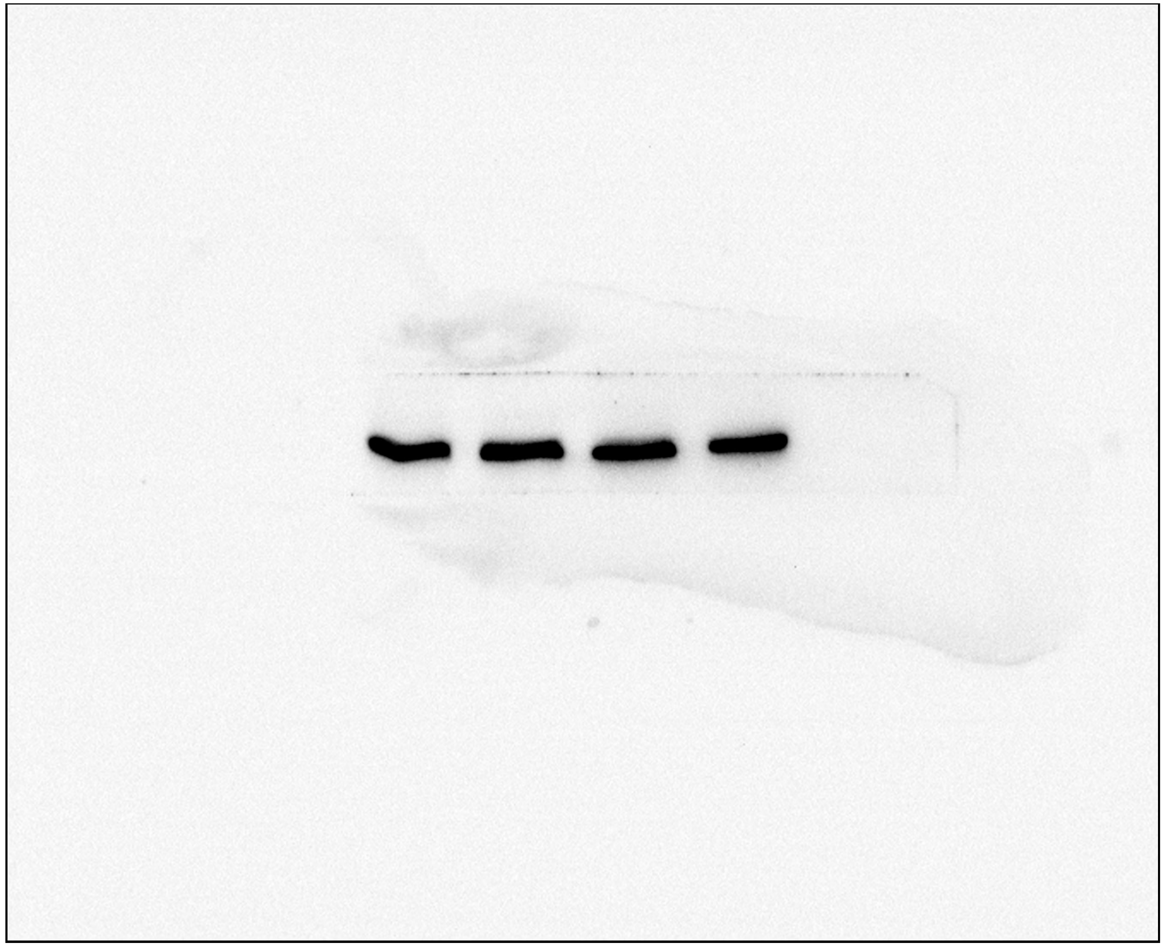

Figure 6E

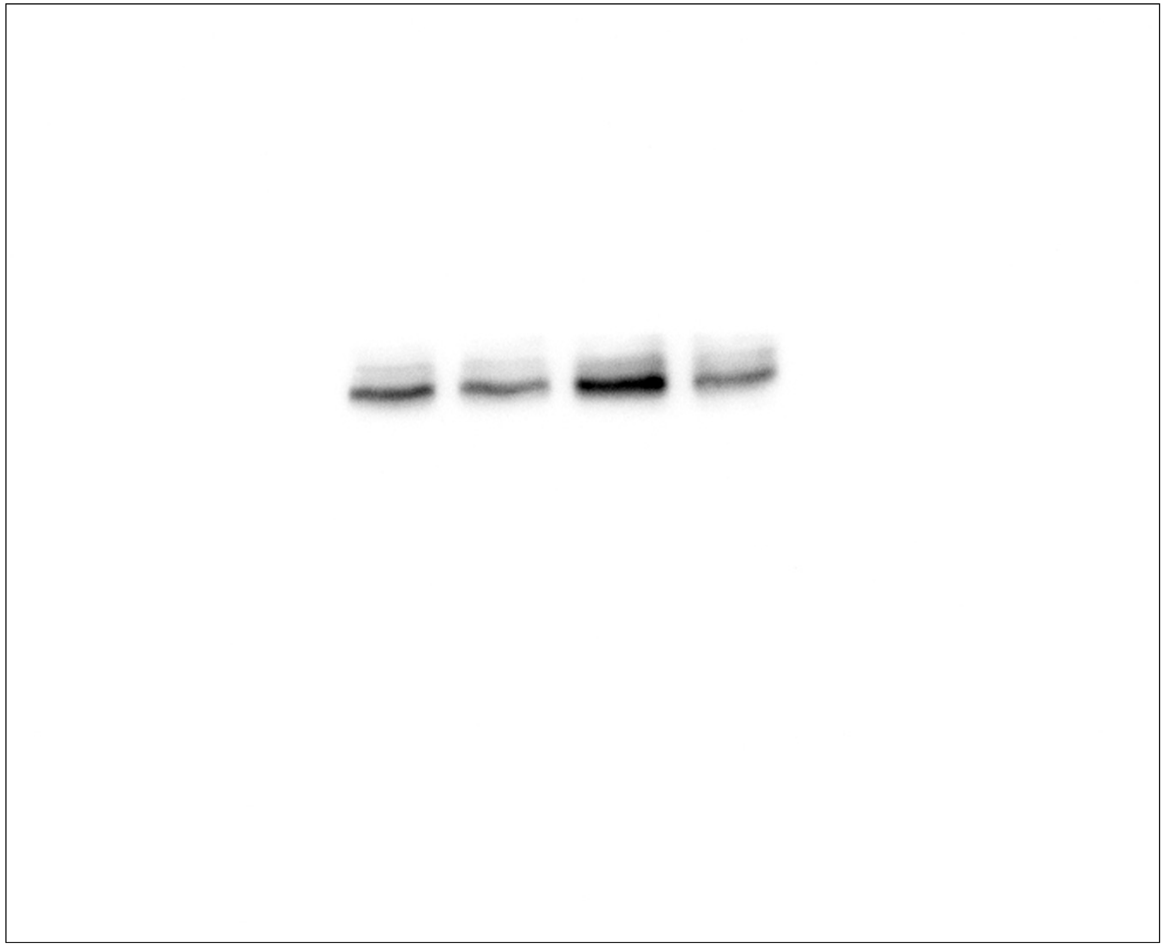

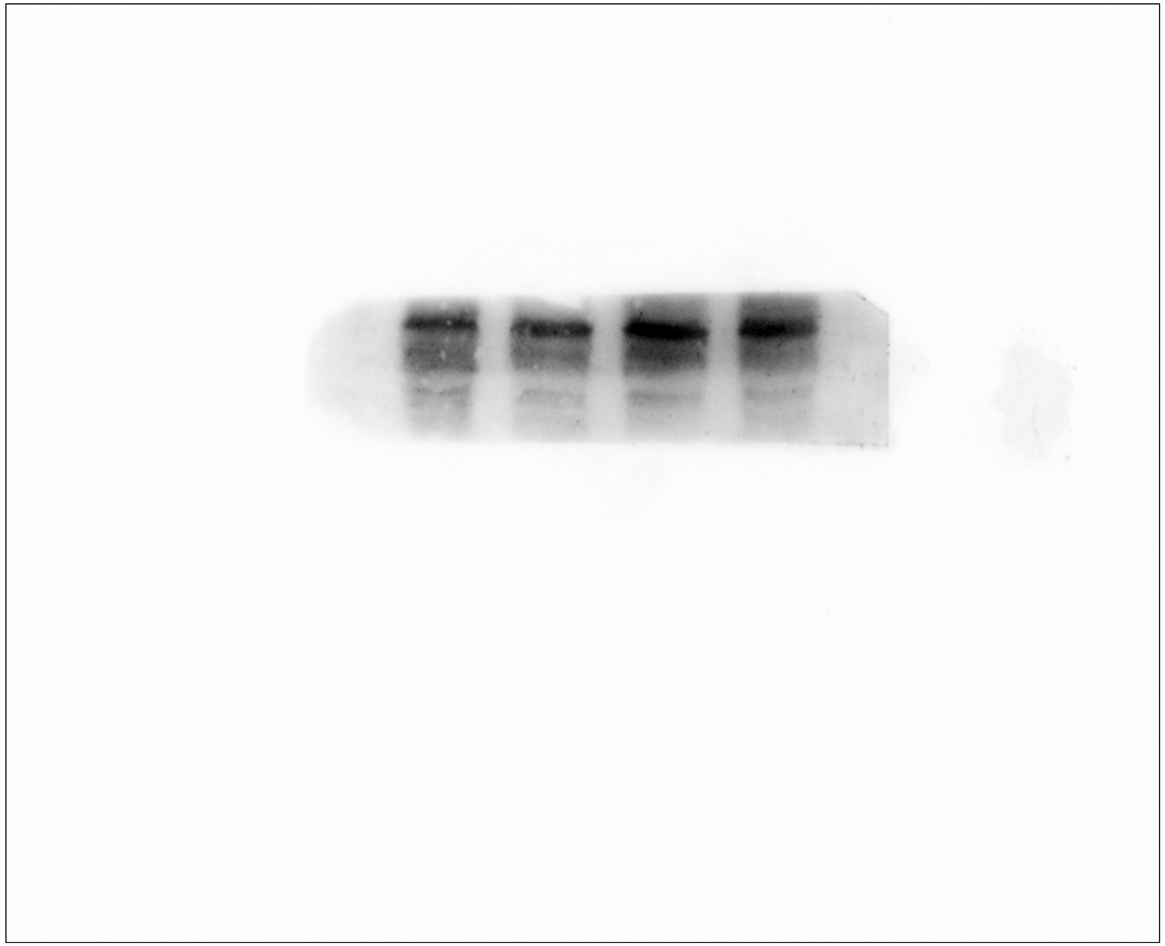

Figure 6E

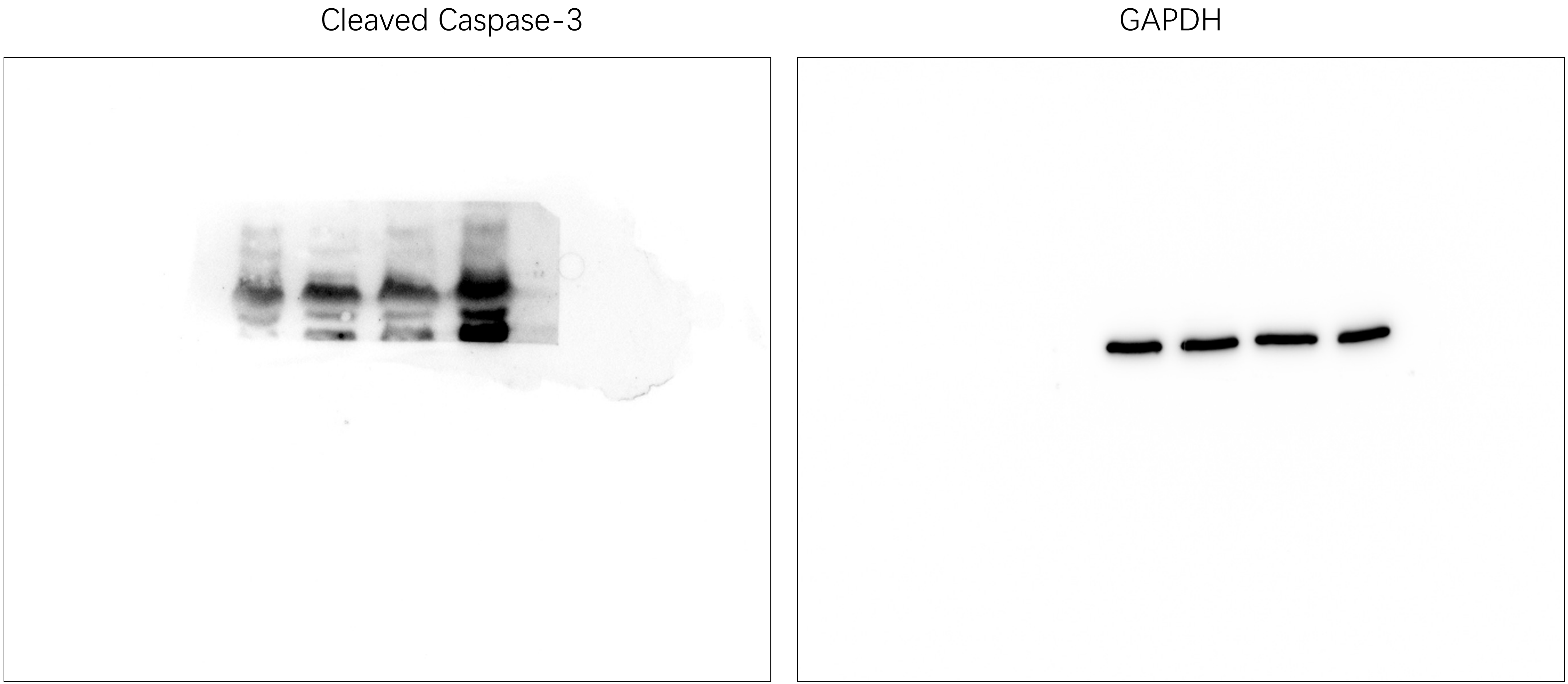

Supplementary figure 3B

KLF5

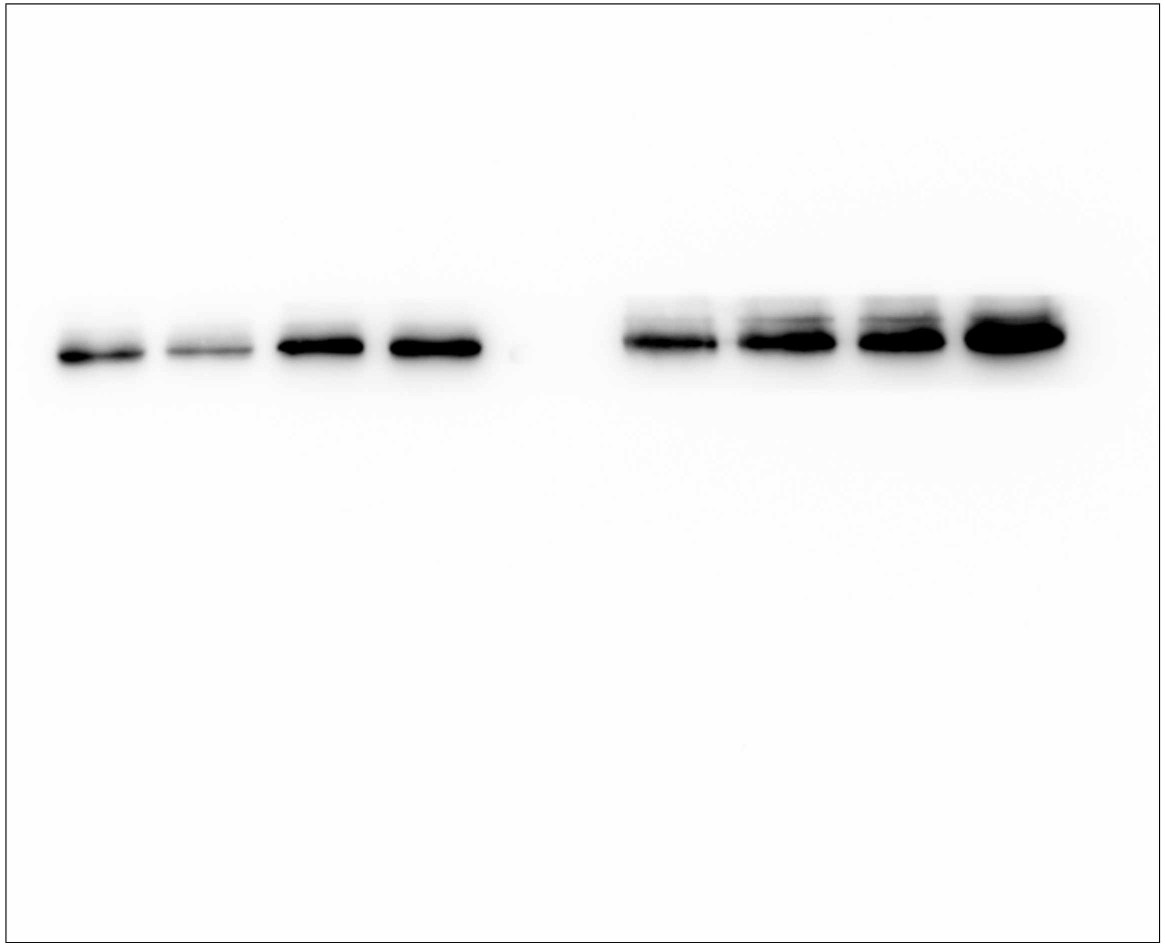

GAPDH

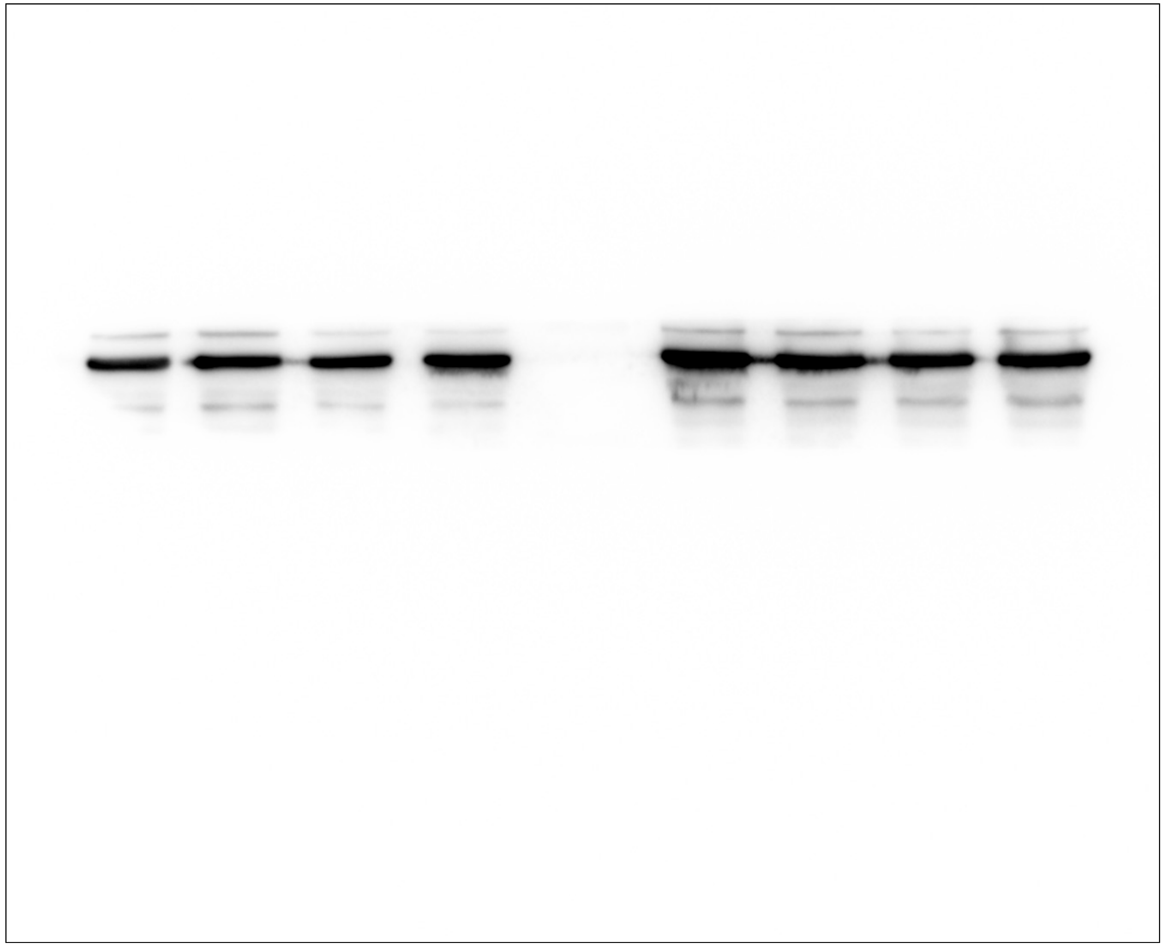

Supplement: Supplementary file 8 — Original Data File [file 41419_2022_4773_MOESM8_ESM.pdf]
